# Irrational risk aversion in an ant

**DOI:** 10.1007/s10071-021-01516-1

**Published:** 2021-05-03

**Authors:** Massimo De Agrò, Daniel Grimwade, Richard Bach, Tomer J. Czaczkes

**Affiliations:** 1 Department of General Psychology, University of Padova, Padova, Italy; 2 Animal Comparative Economics Laboratory, Department of Zoology and Evolutionary Biology, University of Regensburg, Regensburg, Germany; 3 John Harvard Distinguished Science Fellows Program, Harvard University, Cambridge (MA), USA; 4 Department of Organismic and Evolutionary Biology, Harvard University, Cambridge (MA), USA; 5 Department of Zoology, University of Oxford, Oxford, UK

**Keywords:** Risk aversion, Psychophysics, Utility, Value perception, Ants

## Abstract

Animals must often decide between exploiting safe options or risky options with a chance for large gains. Both proximate theories based on perceptual mechanisms, and evolutionary ones based on fitness benefits, have been proposed to explain decisions under risk. Eusocial insects represent a special case of risk sensitivity, as they must often make collective decisions based on resource evaluations from many individuals. Previously, colonies of the ant *Lasius niger* were found to be risk-neutral, but the risk preference of individual foragers was unknown. Here, we tested individual *L. niger* in a risk sensitivity paradigm. Ants were trained to associate one scent with 0.55 M sucrose solution and another with an equal chance of either 0.1 or 1.0 M sucrose. Preference was tested in a Y-maze. Ants were extremely risk-averse, with 91% choosing the safe option. Based on the psychophysical Weber–Fechner law, we predicted that ants evaluate resources depending on their logarithmic difference. To test this hypothesis, we designed 4 more experiments by varying the relative differences between the alternatives, making the risky option less, equally or more valuable than the safe one. Our results support the logarithmic origin of risk aversion in ants, and demonstrate that the behaviour of individual foragers can be a very poor predictor of colony-level behaviour.

**Supplementary Information:**

The online version contains supplementary material available at 10.1007/s10071-021-01516-1.

## Introduction

Traditionally, organisms were assumed to maximise energetic gains while minimising costs, on the basis that evolution should drive animals to have optimal behavioural strategies. However, the optimal foraging theory framework (Pyke et al. 1977) fails to fully describe behaviour—organisms do not always behave optimally. Such deviations are often described as “irrational”, although it is broadly acknowledged that such deviations often follow bounded or ecological rationality (Fawcett et al. 2014). Extensive examples of violation of optimality in animal species can be found, for example, in the literature about risk sensitivity (Caraco et al. 1980). We define risk as a situation in which the probabilities associated with an option (e.g. food source) are known, but the exact payoff which will be received is not known. For example, rolling a 6-sided die hoping for a 1 is a risky proposition, with a known success chance of 1/6. Conversely, “uncertainty” is when not even the probabilities of the various possible payoffs are known. Rolling a die with an unknown number of sides hoping for a 1 is a proposition under uncertainty.

### Risk sensitivity theories—the budget rule

Risk sensitivity studies were effectively inaugurated by Caraco et al. (1980). They studied the preference of yellow-eyed juncos for different amounts of seeds: one of the two alternatives available to the birds was stable, presenting always the same, medium amount of food (safe feeder), while the other one fluctuated in value, but had the same mean pay-out as the safe feeder (risky feeder). The authors then, based on the preference of the animals, designed a utility function (Becker et al. 1964), computing the perceived value (utility) for each number of seeds for the animals. Yellow-eyed juncos presented a concave utility function (and so were risk-averse) when in a high-energy budget, whereas their utility function was convex (and so they were risk-prone) when in a low-energy budget. This behaviour was soon formalized as the Energy Budget Rule (Stephens 1981). The budget rule has recently been reformulated by Lim et al. (2015), who argue that the classical budget rule is often misused in its binomial interpretation: that animals are either risk-prone (when in a low-energy budget) or risk-averse (when in a high-energy budget). However, the optimum risk sensitivity in a given situation lies on a non-linear continuum (Lim et al. 2015): at very low and very high-energy budget risk indifference arises again.

### Proximate explanations for risk sensitivity

It has also been proposed that risk sensitivity arises as a side effect of the neural or cognitive architecture of an animal, or due to evolutionary constraints, and that one need not attempt to fit this behaviour to fitness benefits (Fechner 1860). A striking pattern in risk preference studies is that animals are often risk-averse when risking amounts, but risk-seeking when risking delays (Kacelnik and Bateson 1996). Animals (and humans) are also generally risk-averse for potential gains, but risk-prone for potential losses (Kahneman and Tversky 1979). These patterns are elegantly explained by an understanding of how animals perceive the world, as described by Psychophysics (Gescheider 1976; Tuzlukov 2013; Stevens 2017). Stimulus strength has a logarithmic relationship with perception, as formalized by the Weber–Fechner law (Weber 1834; Fechner 1860). Thus, a constant feeder that always presents 5 seeds and a variable feeder presenting alternatively 1 or 9 seeds have the same average; however, 5 seeds are valued as 5 times more than 1, while 9 is not even twice as good as 5. Thus, while the mathematical average, and so the true energetic value, of the variable feeder is the same as the one of the safe feeder, its geometric average is lower. According to the Weber–Fechner law, which describes how animals perceive stimuli intensity or values, the midpoint of variable source presenting two values is coincident with the geometrical average rather than the mean. Relatedly, Scalar Utility Theory (SUT) was postulated (Marsh and Kacelnik 2002; Kacelnik and El Mouden 2013) to describe risk aversion behaviour. Marsh and Kacelnik point out that, based on the Weber–Fechner law, the variance of the memory representation of a food value increases as the value itself increases. This is because the memory trace for low values (1 in our previous example) remains very precise, while high values (9) becomes fuzzier. The value of a variable food source is equivalent to the median point of the combined memory trace. Due to the different variance of the low and high values, however, two options with identical mathematical average (means) will have different medians, with the more variable option having a lower one (see Fig. 6 from Kacelnik and El Mouden 2013 for a complete explanation). The geometrical average between two values will be coincident with the median of the two memory traces, as the difference in variance is derived from the Weber–Fechner law.

### Ants as a model for risk sensitivity

Risk sensitivity has been studied in a great variety of animals (for a review, see Kacelnik and El Mouden, 2013). Among those, nectarivores have received particular scrutiny (Perez and Waddington 1996; Shafir 2000). The majority of studies on nectarivores have been carried out on bees. Results have, however, been unclear, possibly due to the previously described binomial interpretation of risk (Shafir 2000; Weber et al. 2004): bees have been observed to be risk-indifferent (Banschbach and Waddington 1994; Perez and Waddington 1996; Fülöp and Menzel 2000), risk-averse (Waddington et al. 1981; Shapiro 2000), to follow the budget rule (Cartar and Dill 1990; Cartar 1991), or a mixture of those depending on risk variability (Shafir et al. 1999; Shafir 2000; Mayack and Naug 2011; Dunlap et al. 2017). Bees and other eusocial insects represent a special case for risk sensitivity. For eusocial insects with non-reproductive workers, the colony is the main unit of selection and a colony can be considered a superorganism (Hölldobler and Wilson 2009; Boomsma and Gawne 2018). As such, the foraging successes of the individual workers are pooled. This buffers colonies against short-term (negative) fluctuation coming from risky choices made by individual foragers. Colonies can also visit multiple food sources simultaneously, allowing them to more efficiently exploit their environment (Devigne and Detrain 2005; Czaczkes et al. 2015a). Lastly, many eusocial insects can make collective foraging decisions, using recruitment mechanisms to channel workers towards certain resources in the environment (Detrain and Deneubourg 2008; Gordon 2019).

While research on risk preference and collective decision-making is extensive, these have rarely been combined. Collective risk sensitivity has been explicitly studied in ants. Burns et al. (2016) presented colonies of rock ants (*Temnothorax albipennis*) a fixed-quality mediocre nest and a variable-quality nest. Ants were allowed to explore (and hence evaluate) each nest and then recruit nestmates, and colonies were found to be risk-prone. On the other hand, Hübner and Czaczkes (2017) tested the risk sensitivity of black garden ant (*Lasius niger*) colonies to food values. Each colony was presented with two feeders: a stable one, always presenting the same, medium-quality sucrose solution (0.55 M), and a variable one, presenting alternatively (changing every 3 min) either low- or high-quality sucrose solution (0.1 M–1.0 M). Almost all trials showed a clear collective decision for one of the two feeders (as is expected due to symmetry breaking in ants’ collective decisions, see Beckers et al. 1990, 1993; Czaczkes et al. 2015b; Price et al. 2016) but, overall, colonies were risk-indifferent: half the colonies chose the safe feeder, and half chose the risky one, regardless of whether it began by offering the high or the low reward. This is surprising, as positive feedback from the initially best food source should have resulted in symmetry breaking and a collective choice for the initial feeder offering the highest reward (Beckers et al. 1993; Detrain and Deneubourg 2008; Czaczkes et al. 2015b; Price et al. 2016).

The current work aims to explore individual risk preference in individual *Lasius niger* ant foragers. Although their collective behaviour appears to be rational in terms of the absolute sugar amount retrieved, individual workers may not be (Sasaki and Pratt 2011; Sasaki et al. 2019). They could be subjected to the same perceptual constraints discussed above and be strongly influenced by expectations (Wendt et al. 2019), causing rejection for some food alternatives, triggering risk aversiveness.

## Materials and methods

### Subjects

Twenty-seven queenless *Lasius niger* colony fragments, consisting of around 1000 ants each, were used in the experiment. Each fragment was collected from a different wild colony on the University of Regensburg campus. Workers from colony fragments forage, deposit pheromone and learn well (Evison et al. 2008; Oberhauser et al. 2018). Each fragment was housed in a transparent plastic box (30 × 20 × 40cm), with a layer of plaster on the bottom. A circular plaster nest, 14 cm in diameter and 2 cm thick, was also provided. The colonies were kept at room temperature (21–25 °C) and humidity (45–55%), on 12:12 light: dark cycle. Each colony was fed exclusively on 0.5 M sucrose solution ad libitum, and deprived of food 4 days prior to each test. Water was provided ad libitum and was always present.

### Experiment 1 – Risk preference between options of equal energetic value

The aim of this experiment was to assess the preference of individual ants between two food sources which provide, on average, an equal amount of sucrose: one feeder provided a stable moderate value (0.55 M sucrose, the ‘safe’ option) and one provided a fluctuating value, either high or low (0.1 M or 1.0 M, the ‘risky’ option). This was achieved by teaching each individual ant to associate each feeder type (risky or safe) with a different odour, and then testing their preference in a Y-maze. Preliminary tests (see ESM1) and previous work (Czaczkes et al. 2018a, b) show that *L. niger* foragers learn quickly (within 3 visits to each odour) and reliably to associate odours with feeders of different types. In total we tested 64 ants equally divided among 4 different colonies.

#### Training

To begin each experiment, ants were allowed onto a 15 cm long, 1 cm wide runway, with a drop of sucrose at the end. Each drop was big enough to allow the ant to drink to satiation without consuming it completely. The first ant to encounter the sucrose was marked with a dot of paint, and all other ants were returned to the nest. The marked ant was allowed to drink to satiety and then return to the nest to unload the collected sugar. She was then allowed to make 7 further training visits to the runway and feeder. In each visit, we recorded the number of pheromone depositions performed on the runway towards the feeder and towards the nest after foraging. Over the 8 visits, the quality and odour of the feeder were alternated so that the ant encountered, for example, first a moderate-quality drop of sucrose solution (0.55 M, ‘safe’) scented with one odour, then a low (0.1 M) valued drop (‘risky’) scented with another odour, then the safe option with the first odour again, then a highly (1.0 M) valued drop (‘risky’) with the second odour. These values are clearly distinguishable by the ants (Wendt et al. 2019) and correspond to moderate-, low-, and high-value food sources for *L. niger* (Detrain and Prieur 2014). Ants were thus conditioned to associate two odours with two different qualities of food. One odour was associated with a fixed quality (safe option) and the other odour was associated with two alternating qualities (risky option). Note that the average of the low- and high-quality solutions equals that of the moderate-quality. The solutions were scented using either rosemary or lemon essential oils (0.05 µl per ml). The runway leading to the feeder was covered with a paper overlay scented identically to the sucrose solution being offered. Overlays were scented by storing them in a sealed box containing cotton soaked in essential oil. Overlays were discarded after each return to the nest, to ensure fresh odour and to prevent a build-up of trail pheromone from occurring.

For each tested ant, one odour corresponded to the ‘risky’ feeder and one to the ‘safe’ feeder. The experiments were balanced for each ant with respect to all of the parameters (odour of safe and risky, first odour presented, first risk alternative presented, odour side on the Y-maze). Performing treatments blind was attempted, but due to the clear negative contrast effects shown by ants on encountering a low-quality food source after better ones (Wendt et al. 2019), true blinding was not possible.

#### Testing

After the 8 training visits, the runway was replaced with a Y-maze (arm length 10 cm, bifurcation angle 120°). The stem of the Y-maze was overlaid with unscented paper, whereas the two other arms were covered with scented overlays, one bearing the ‘risky’ associated scent, and the other the ‘safe’ associated scent. The maze tapered at the bifurcation to ensure that the ant perceives both scented arms at the same time (following Czaczkes 2018). No sucrose was present on the Y-maze. We recorded the ants’ initial arm decision, defined by the ants’ antennae crossing a line 2 cm from the bifurcation point. We also recorded the ants’ final decision, defined by the ant crossing a line 8 cm from the bifurcation point. However, the initial and final decisions of the ants were almost always the same, and analysis of either choice provides the same results (see ESM2). For brevity we henceforth discuss only the initial decision data. On reaching the end of an arm the ant was allowed to walk onto a piece of paper and brought back to the start of the Y-maze stem, to be retested. The Y-maze test was thus repeated 3 times, which we expected initially to be capable of assessing reliability of the ant choice. However, we observed that this handling may have caused some disruption (see ESM2) and repeated unrewarded trials affect motivation, so we conservatively analysed only the first Y-maze test. After testing, the ant was permanently removed from the colony.

### Experiment 2 – Risk preference between options of different absolute value

Experiment 1 demonstrated very strong risk aversion in individual ant foragers. Experiment 2 was designed to test whether risk aversion would be maintained ‘irrationally’, that is, when the ‘risky’ feeder had an objectively higher average quality than the ‘safe’ feeder.

As in experiment 1, the ‘safe’ feeder always presented a medium-quality drop (0.55 M). However, the ‘risky’ feeder alternated between a low-quality reward (0.1 M) and a very high-quality reward (1.5 M). The average molarity of the risky feeder (0.8 M) was thus higher than the average molarity of the safe one. *L. niger* foragers can distinguish between the three presented molarities (Wendt et al. 2019). Moreover, in a pilot experiment, we observed that when presented with three different molarities ants do learn all three molarities and their associated odours (see ESM2, Czaczkes and Kumar 2020). Each ant was tested on the Y-maze 5 times, but as in experiment 1, only data from the first test were ultimately used (see ESM2). In total, we tested 64 ants from 8 colonies. Each condition (scent association, feeder order, risky feeder order, scent side on the Y-maze) was balanced and equally distributed among colonies.

### Experiment 3 – Risk preference between psychophysically balanced options

One hypothesis explaining the widespread risk aversion found in animals towards reward quantities arises from the psychophysics of perception: intensity is generally perceived logarithmically (Kacelnik and Bateson 1996; Kacelnik and El Mouden 2013; see introduction). It is thus the geometrical average between the two risky alternatives that may describe the perceived value. This hypothesis predicts that animals should be indifferent between a safe and a risky option, if the two are balanced in respect to a logarithmic curve. In experiment 2, these were not balanced: the geometrical average of the risky feeder $\left( {\sqrt {0.1 \times 1.5} = 0.{387}} \right)$ was still lower than the one of the safe feeder $\left( {\sqrt[1]{{0.{55}}} = 0.{55}} \right)$; thus, the risky option may still have been perceived as worse than the safe option. In this experiment, we set out to offer a ‘risky’ option in which the *perceived* qualities of the low and high reward were balanced relative to the moderate reward. We chose a moderate reward of 0.3 M, and a low and high reward of 0.1 M and 0.9 M, respectively. The geometrical average of the risky option $\left( {\sqrt {0.1 \times 0.9} = 0.3} \right)$ was now equal to the one of the safe option. We thus hypothesised that ants would be indifferent between these two options. Each ant was tested on the Y-maze 5 times, but again only data from the first test was used (see ESM2). In total, we tested 40 ants from 10 different colonies. Each condition (scent association, feeder order, risky feeder order, scent side in the Y-maze) was balanced and equally distributed among colonies.

### Experiment 4 – Risk preference with a psychophysically higher-valued risky alternative

To confirm the hypothesized logarithmic curve of perceived value, we designed a 4th experiment, in which the risky option would present a higher value in term of the molar geometrical average in respect to the safe alternative. However, this presented significant challenges: the three molarities presented must be easily distinguishable by the ants, and must lie in a specific range to be accepted by the animals. Moreover, the geometrical average of the risky alternative should be perceptibly higher than the safe value, to observe a statistically significant preference. For this experiment, we used 0.5 M for the ‘safe’ option, 0.25 M and 2.0 M for the low and high alternatives of the ‘risky’ one. With these alternatives, the geometrical average of the ‘risky’ feeder $\left( {\sqrt {0.25 \times 2.0} = 0.707} \right)$ was higher than the ‘safe’ one. However, it is worth noting that ants value perception may plateau for molarities above 1.5, as previous literature suggests (Wendt et al. 2019), with all molarities above 1.5 considered equally valuable. This would cause the perceived value of the ‘risky’ feeder $\left( {\sqrt {0.25 \times 1.5} = 0.612} \right)$ to be just slightly higher than the safe alternative. In total we tested 64 ants from 5 different colonies.

### Experiment 5 – Risk preference with an absolutely higher-valued risky alternative

Lastly, we tested the ants’ ability to actually remember a variable food source, and prefer it to a constant one. In this condition, the ‘risky’ alternative presented an absolutely better payoff over the ‘safe’ one, as the low alternative of the risky option was equal to the safe option. In this experiment, there is actually no risk associated with choosing the variable food source, which will still be called ‘risky’ for consistency with the other experiments. Specifically, the low and high values for the ‘risky’ feeder were 0.25 M and 1.5 M, respectively, while the value of the ‘safe’ feeder was 0.25 M. In this condition, if ants have no absolute rejection for variable food source, or a mnemonic limitation for such conditions, we expected the ants to choose the ‘risky’ option. We tested in total 64 ants coming from 5 different colonies.

### Statistical analysis

Statistical analyses were carried out in R 4.0.5 (R Core Team 2020). Following Forstmeier and Schielzeth (2011), we included in the models only factors and interactions for which we had a priori reasons for including. We employed generalized linear mixed-effect models using the package lme4 (Bates et al. 2015), with colonies as a random effect. Y-maze choice data were modelled using a binomial distribution and logit link function. We used the following model:

$$ \begin{gathered} {\text{Initial decision }} = \hfill \\ {\text{first presented feeder}}\left( {{\text{risky}} - {\text{safe}}} \right)* \hfill \\ {\text{first presented risky alternative }}\left( {{\text{good}} - {\text{bad}}} \right) \, + \hfill \\ {\text{random effect }}\left( {{\text{colony}}} \right) \hfill \\ \end{gathered} $$

We then used the package car (Fox and Weisberg 2011) to test which factors of the model had a significant effect on the dependent variable. Subsequently, we carried out post hoc analysis with Bonferroni correction using the package emmeans (Lenth 2018) both for the general preference of the ants for either the safe or the risky feeder (safe choice probability against random probability), and for the factors with a significant effect to analyse the direction of the difference. Plots were generated using the package ggplot2 (Wickham 2009) and the python (Van Rossum and Drake 2009) matplotlib library (Hunter 2007).

While we collected and analysed a wealth of pheromone deposition data (see ESM1, 2, 3), for brevity, we omit reporting on this data. We warmly encourage interested readers to examine, analyse, and use this data in any way they see fit.

Only the main results are reported below. For the full analysis see ESM2. The raw data for all the experiments can be found in the supplemental materials ESM3.

## Results

### Experiment 1 – Risk preference between options of equal energetic value

**Fig. 1:**
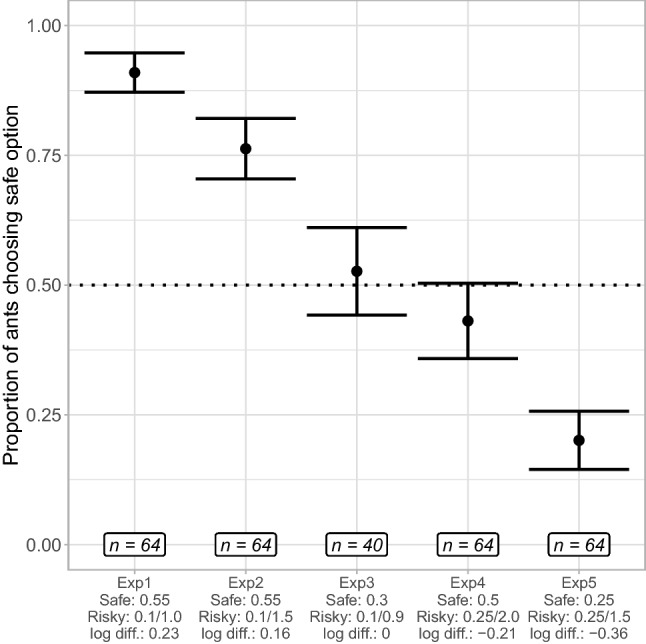
Proportion of ants choosing the safe feeder; error bars represent standard error. Ants’ preference is different from chance level in experiment 1 (prob. = 0.911, SE = 0.36, z ratio = 5.142, *p*-value < 0.0001), in experiment 2 (prob. = 0.792, SE = 0.068, z ratio = 3.248, *p*-value = 0.001) and in experiment 5 (prob. = 0.18, SE = 0.053, z =  − 4.182, *p* < 0.0001), but not in experiment 3 (prob. = 0.535, SE = 0.086, z ratio = 0.403, *p*-value = 0.687) or experiment 4 (prob. = 0.427, SE = 0.085, z =  − 0.844, *p* = 0.398)

Ants were strongly risk-averse, with 91% (58/64) ants initially choosing the safe option (Fig. 1) (GLMM post hoc with estimated means, probability = 0.911, SE = 0.036, z = 5.142, *p *< 0.0001). We found no effect of the first presented feeder (GLMM Analysis of Deviance, Chi-square = 0.709, DF = 1, *p* = 0.3), nor of the first presented risky alternative (Chi-square = 0, DF = 1, *p* = 1), nor of the interaction between those two factors (Chi-square = 0, DF = 1, *p* = 1).

### Experiment 2 – Risk preference between options of different absolute value

Ants were again strongly risk-averse, with 75% (48/64) ants initially choosing the safe option (Fig. 1) (GLMM post hoc with estimated means, probability = 0.792, SE = 0.068, z = 3.248, *p* = 0.001). We found no effect of the first presented feeder (GLMM Analysis of Deviance, Chi-square = 2.015, DF = 1, *p* = 0.156), nor of the first presented risky alternative (Chi-square = 0.197, DF = 1, *p* = 0.657), nor of the interaction between those two factors (Chi-square = 1.807, DF = 1, *p* = 0.179).

### Experiment 3 – Risk preference between psychophysically balanced options

53% (21/40) of ants chose the safe option (Fig. 1), a proportion not different from chance (GLMM post hoc with estimated means, probability = 0.535, SE = 0.086, z = 0.403, *p* = 0.687).

We found an effect of the first presented feeder (GLMM Analysis of Deviance, Chi-square = 4.424, DF = 1, *p* = 0.0354). Specifically, 71% of the ants that were presented with the safe feeder in visit 1 choose the safe smell during testing, while 35% of the ones presented with the risky feeder first did.

### Experiment 4 – Risk preference with a psychophysically higher-valued risky alternative

44% of the ants (28/64) chose the safe option, a percentage not different from chance level (Fig. 1) (GLMM post hoc with estimated means, probability = 0.427, SE = 0.085, z =  − 0.844, *p* = 0.398). We also found an effect of the first presented feeder (GLMM Analysis of Deviance, Chi-square = 5.4, DF = 1, *p* = 0.02); specifically, more ants chose the safe alternative in the test if they had experienced it first in the training (GLMM post hoc with estimated means, odds ratio = 0.266, SE = 0.148, z =  − 2.38, *p* = 0.017).

### Experiment 5 – Risk preference with an absolutely higher-valued risky alternative

Only 20% of the ants (13/64) chose the safe option, a percentage significantly lower than chance level (Fig. 1) (GLMM post hoc with estimated means, probability = 0.18, SE = 0.053, z =  − 4.182, *p* < 0.0001). We found no effect of the first presented feeder (GLMM Analysis of Deviance, Chi-square = 0.1, DF = 1, *p* = 0.751), the first value of the risky option (Chi-square = 2.468, DF = 1, *p* = 0.116) nor the interaction between the two (Chi-square = 1.244, DF = 1,* p* = 0.2647).

## Discussion

Ants show strong risk aversion given equal average payoffs between the risky and safe options (0.1/1.0 M vs. 0.55 M, experiment 1). Even if the risky option offers 45% higher mean payoffs than the safe reward (0.1 M/1.5 M vs. 0.55 M), ants still show strong risk aversion (experiment 2). We predicted, based on psychophysical principles, that logarithmically balanced rewards should be perceived as having equal value. We tested this in a situation where the risky reward offered 66% higher payoffs than the safe reward (0.1/0.9 M vs 0.3 M) and observed, as predicted, indifference between the two options (experiment 3). When presented with a higher-valued alternative according to the logarithmic perception of value (0.25 M/2.0 M vs. 0.5 M, experiment 4), ants showed a higher probability of choosing the risky option, but not at a level significantly over chance. When presented with an absolutely better risky alternative (0.25 M/1.5 M vs. 0.25 M), the ants chose consistently the risky option (experiment 5).

**Fig. 2:**
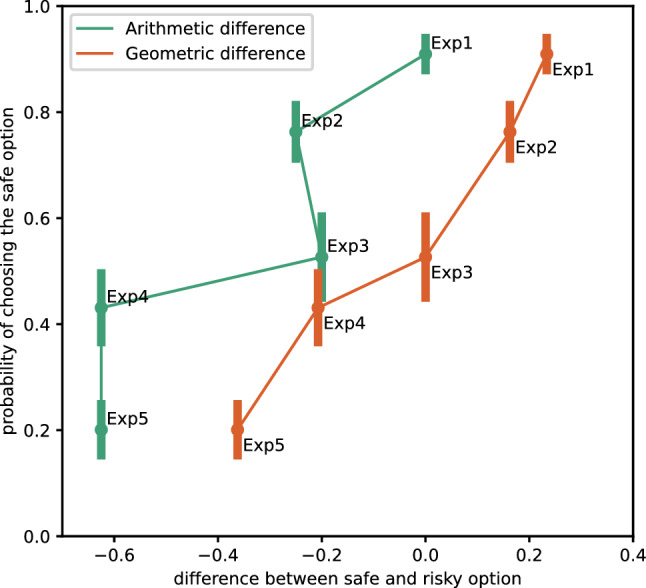
Proportion of ants choosing the safe feeder ordered by the difference between the safe and the risky feeder values, as calculated based on the arithmetic average and the geometrical average. Crucially, the preference for the safe feeder scales fairly linearly when considering the geometrical average, while the arithmetical average shows no discernable pattern. Moreover, the geometrical model resulted in a significantly better fit than the arithmetical one (Vuong test for non-nested glm, z = 2.003, *p* = 0.02257

The ants’ lack of a significant preference in experiment 4 went against our expectation. As discussed in the methods section, however, the molarity levels that can be used in such an experiment are limited by the ants’ range of acceptance. It is possible (and in line with previous literature, see Wendt et al. 2019), that values above 1.5 M are regarded as equal by the animals, since the costs associated with denser rewards (e.g. speed of consumption) balance out the gains (Lois-Milevicich et al. 2021). If in experiment 4 the ants considered the high-value alternative as 1.5 M, the resulting geometrical average $\left( {\sqrt {0.25 \times 1.5} = 0.612} \right)$ would result only slightly higher than the safe alternative, which would be in line with the marginal preference we observed. Regardless, ants can clearly learn and choose a variable food source, as shown by experiment 5. To cement the geometrical average hypothesis, we also modelled the ants’ preference for the safe feeder according to the geometrical and arithmetical average difference between alternatives. Then, we ran a Vuong model selection (Vuong 1989; Merkle et al. 2016) to test which of the two (geometrical or arithmetical) was better. The geometrical average difference was found to be a significantly better predictor (z = 2.003, *p* = 0.02257, Fig. 2).

Our demonstration of risk aversion in resource amounts is in line with the perceptual, descriptive theory of risk sensitivity proposed by Kacelnik & Bateson (1996) and developed by Kacelnik & El Mouden (2013). Specifically, our data are consistent with functional risk aversion arising from risk neutrality filtered through logarithmic perception. It is striking that we were able to accurately predict an indifference point for the ants solely on the basis of basic psychophysical principles. However, our results are also consistent with Budget Rule theories (Stephens 1981), since the ants are on a positive energy budget—*Lasius niger* would survive for over a week without feeding.

### Lack of support for Prospect Theory

Other theories of risk sensitivity based on perceptual mechanisms exist, in addition to Scalar Utility Theory. Prospect Theory (Kahneman and Tversky 1979), a hugely influential economic theory of decision-making under risk in humans, predicts that an individual should be risk-averse in the context of gains but risk-prone in the context of losses. This again derives from logarithmic perception of cumulative gains and losses. However, in Prospect Theory, the dividing point between gains and losses is not necessarily at zero. Rather, gains and losses are defined relative to a reference point, which is usually the expected payoff, but may be socially induced (e.g. by comparing one’s own salary to that of one’s colleagues). Anything above the reference point is perceived as a gain and anything below the reference point is a loss. Rejection of a lower value after a reference has been established has already been demonstrated in the honeybee (Couvillon and Bitterman 1984) and ants (Wendt et al. 2019), and suggested in bumblebees (Wiegmann et al. 2003). The reference point for our colonies might have been 0.5 M: the solution that the ants are regularly fed on. If this were the case, in experiment 1, the true choice would be between an always neutral value (0.55 M, safe), and a risk between a gain (1.0 M) and a loss (0.1 M). This hypothesis is also supported by the fact that almost no pheromone was deposited for the 0.1 M drop, suggesting that it may have been perceived as a loss. In this case, Prospect Theory would still predict risk aversion, as losses are assumed to be perceived more strongly than gains. To test this hypothesis, we repeated experiment 1, but with colonies that had been fed ad libitum 1.5 M sucrose 1 month prior testing (data and procedure can be found in ESM1). If the ants were taking their standard feeding solution as a reference point, every presented solution in this experiment should have been perceived as a loss, and so the ants should have shown risk-seeking. However, we observed the same preference that we saw in the main first experiment, strong risk aversion. Either the ant’s behaviour is poorly described by Prospect Theory, or the normal feeding solution does not set the reference point. Another possibility is that the reference point is not set by the normal feeding solution, as the four-day food deprivation period may erase the ant’s memory of the feeding solution (although learned associations last at least three days in other species, Piqueret et al. 2019). Instead, the reference point could be the most common solution in the current context. In experiment 1, this would be 0.55 M, maintaining the same situation of one neutral vs. a loss or a gain, and so predicting the same outcome under Prospect Theory. This hypothesis, however, does not fit the result obtained in experiment 3: if the 0.3 M would have been taken as a reference, we should still have observed a preference for the safe option. Either Prospect Theory does not well describe the behaviour of ants, or their reference point remains at 0 in every situation, with every reward being a gain: in the domain of gains Prospect Theory predicts simple logarithmic value perception.

In this study, we found individual ants to be strongly risk-averse, and successfully predicted an indifference point for risk based on the psychophysical principles of perception. Individual risk preference does not predict colony behaviour (Hübner and Czaczkes 2017), which seems able to filter out perceptual biases (Sasaki and Pratt 2018).

## Supplementary Information

Below is the link to the electronic supplementary material.

Supplementary file1 (PDF 238 KB)

Supplementary file2 (HTML 4241 KB)

Supplementary file3 (XLSX 595 KB)
