# Supplementary material for: Irrational risk aversion in an ant
Source: Anim Cogn. 2021 May 3;24(6):1237–45. doi: 10.1007/s10071-021-01516-1 (PMC8492575; doi:10.1007/s10071-021-01516-1)
Supplement: Supplementary file 1 — Supplementary file1 (PDF 238 KB) [file 10071_2021_1516_MOESM1_ESM.pdf]

Supplemental Material 1

Table of Contents

1 Control experiments....................2
1.1 Ant perception of 0.1, 0.3 and 0.9 sucrose molarities....................2
1.2 Ant preference among 3 molarities....................4
2 Risk preference in the context of losses (maintained on 1.5M sucrose)....................5
3 Pheromone deposition data....................5
Discussion....................8
4 References....................10

1 Control experiments

1.1 Ant perception of 0.1, 0.3 and 0.9 sucrose molarities

In experiment 3, the ants were presented with feeders offering 0.1, 0.3 and 0.9 molar sucrose. Relative to experiments 1 and 2, the medium- and the low-quality drop had very similar molarities in absolute terms, so that we decided to run a pilot experiment to test whether the ant could discriminate, and subsequently choose reliably between, the three molarities.

We ran two testing blocks. In the first, the ants were presented with two drops of different molarities, 0.1 and 0.3, and were trained to associate each to a smell. We followed the methodology described for the main experiment (see methods section in the main paper), alternating the presentation of the low-quality alternative and the high-quality alternative in the 8 training visits. Afterwards we tested the ants in the Y-maze, repeating the test 5 times. The second experiment was identical to the first, but the ants were presented with 0.3 and 0.9 molarities. We did this last block just as a control, since as 0.3 and 0.9 are further apart than 0.55 and 1.0 we were confident that they could discriminate between the two. We tested 20 ants for each block (40 in total), stemming from 6 different colonies. First, we tested the robustness of the ants' choices, checking whether with subsequent visits the number of ants choosing the high value drop decreased. We modelled as follows:

High value choice (all tests) =
Testing visit (1-5)+
Contrast (0.1vs0.3 or 0.3vs0.9)
random effect (individual ant nested in colony)

We found that the ants did not change their preference over subsequent visits for either of the two contrasts, differently from what we observed in the main experiment (table S1). This could be because comparing two malarities is easier than the risk-vs-safe evaluation. For the subsequent analysis, we kept all 5 testing visits. We modelled the data as follows:

High value choice (all tests) =
Decision line+
Contrast (0.1vs0.3 or 0.3vs0.9)
random effect (individual ant nested in colony)

Then, we ran a post-hoc test to check which of the groups differed from chance level. We found that the ants significantly preferred 0.3 over 0.1 when considering both the first decision line and the second decision line. However, we found that the ants did not significantly prefer 0.9 over 0.3, remaining at chance level (Table S2). This was surprising to us, as the contrast between 0.9 and 0.3 should be easier to sense, or at least equally difficult if the ants follow the Weber-Fechner law, and in both cases easier than the contrast between 0.55 and 1.0. We suspect that, due to the lower sample size in these experiments, we have experienced a type II error (false negative). However, we decided to present our data as it is, without a post-hoc increase in sample size, following good scientific practice.

Factor	Chi-square	Degrees of freedom	p-value
Contrast	3.5857	1	0.058
Testing visit	1.46	1	0.227
Contrast:Testing visit	0.024	1	0.876

Table S1 – Analysis of deviance (Type II chi-square test) of the model to check difference between testing visit. For this model we had to drop colony as random factor because the model did not converge otherwise. Note that both Testing visit and the interaction between Testing visit and the contrast are not significant. We can conclude that there is no difference between the test visits and there is no difference between the two contrast in testing visits change.

Contrast	Decision line	probability	Standard Error	Z ratio	p-value
0.1 vs 0.3	First decision line	0.86	0.057	3.844	0.0005
0.1 vs 0.3	Last decision line	0.87	0.054	3.988	0.0003
0.3 vs 0.9	First decision line	0.648	0.095	1.461	0.576
0.3 vs 0.9	Last decision line	0.724	0.085	2.264	0.094

Table S2 – post-hoc analysis of the probability of ants choosing the high value alternative, bonferroni corrected.

1.2 Ant preference among 3 molarities

In the main experiment the ants were presented with three different food qualities, and were required to remember all three in order to make a choice between the two feeders. We decided to run a pilot experiment on order to test whether the ants could remember three molarities, rather than just the best one among others.

The ants performed 9 sequential visits to a runway, identical to the one of the main experiment. At the end of the runway the ant may find either a 1.5M drop, always unscented, a 1.0M drop, either rosemary or lemon scented, and a 0.25M drop, scented with the other odour (see table S3).

Visit 1	Visit 2	Visit 3	Visit 4	Visit 5	Visit 6	Visit 7	Visit 8	Visit 9
1.5M	1.0M	0.25M	1.5M	1.0M	0.25M	1.5M	1.0M	0.25M
1.5M	0.25M	1.0M	1.5M	0.25M	1.0M	1.5M	0.25M	1.0M

Table S3 – Training visit sequence for the three molarity experiment. The two rows represent the two possible patterns for the reward presentation. Each ant would experience only one.

Afterwards we tested the ant preference between the 1.0M scent and the 0.25M scent in the Y-maze, repeating the test 5 times. If the ants could only learn the best alternative among the presented ones, they should choose randomly between the second best and the worst. However, if the ants can remember and compare all three values, they should prefer the 1.0M.

We planned to test 32 ants coming from 8 different colonies. However, after having tested 15 ants coming from 5 different colonies we decided to stop the pilot, given the clear preference of the animals: On the first trial, both initial and final decision, 100% of the ants choose the scent associated with 1.0M. We observed a decrease on the ants performance in subsequent files, but the the overall percentage remained at 92%. While we are aware that stopping an experiment prematurely when results are as expected can lead to type I errors (false positives), we felt that the unambiguous nature of these results warranted doing so here.

2 Risk preference in the context of losses (maintained on 1.5M sucrose)

Prospect Theory predicts that individuals should be risk averse in the context of gains and risk prone in the context of losses. The reference point from which we decide if something is a gain or a loss is not necessarily 0: we may take an expected value as a reference. For ants, this value it could be the feeding solution they are maintained on, normally 0.5M. We decided to replicate experiment 1 (see main paper) with 4 colonies that had been fed *ad libitum* 1.5M sucrose instead of the usual 0.5M for one month prior testing. 63 ants were tested in total. Training and testing procedure were identical to those described in the main paper. We found that 82% (52/63) of the ants preferred the safe alternative. All data and analysis are provided in supplement S2

3 Pheromone deposition data

Pheromone deposition count was modelled using a poisson distribution and logit link function. Good model fit was confirmed using the DHARMa package (Hartig 2018), and the pscl package (Zeileis et al. 2008; Jackman 2017) was used to produce the zero-inflated poisson models when needed. Pheromone deposition was not the focus of the current study, but we include it as descriptive data since it may shed light the relationship between individual risk perception and collective decision. We modelled pheromone deposited towards the nest and pheromone deposited on the way back separately, since these are conceptually very different: depositions towards the food reflect the ants' expectation, and depositions on the return to the nest reflect the ants' perception. The models used were the following:

Pheromone towards the drop =

*visit (2-8)**

value (molarity)+

random effect (ant nested in colony)

Pheromone back to the nest =

*visit (1-8)**

value (molarity)+

random effect (ant nested in colony)

Pheromone deposition data from each of the five experiments were analysed separately, as they were taken by three separate experimenters, and so could not reliably be compared between experiments. Path choice decisions allow much less observer error, so Y-maze data can be pooled between experiments.

Experiment 1 – Risk preference between options of equal absolute value

Considering pheromone deposition towards the feeder, we found an effect of molarity (GLMM Analysis of Deviance, Chi square=12.992, DF=2, p=0.001) and an effect of the interaction between molarity and visit number (GLMM Analysis of Deviance, Chi square=14.469, DF=2, p=0.0007). Specifically, we found that the ants deposited overall more pheromone when going towards the 0.55M drop in comparison to the 1.0M drop (figure S4A, GLMM post-hoc with estimated means, estimate=0.657, SE=0.227, z=2.891, p=0.015). Note that the ant may be expecting to find the 0.1M drop when going towards the 1.0M, because it last experienced the low value associated with that scent. Note that this will be the case also for the other experiments. We found no differences in pheromone deposition between the other molarities. Overall, the ants deposited more pheromone on the way to the safe feeder relative to the risky one (GLMM post-hoc with estimated means, estimate=0.498, SE=0.19, z=2.616, p=0.036).

Considering pheromone deposited when returning to the nest, we found an effect of molarity (GLMM Analysis of Deviance, Chi square=85.97, DF=2, p<0.0001), an effect of visit (GLMM Analysis of Deviance, Chi square=5.11, DF=1, p=0.024), but no effect of their interaction. Specifically, we found that the ants deposited overall less pheromone when going back from the 0.1M drop in comparison to the 0.55M drop (figure S4D, GLMM post-hoc with estimated means, estimate=−2.67, SE=0.154, z=−17.352, p<0.0001) and from the 0.1M drop in comparison to the 1.0M drop (GLMM post-hoc with estimated means, estimate=−2.78, SE=0.194, z=−14.308, p<0.0001). However, there was no difference between the 0.55M drop and the 1.0M drop. Overall the ants deposited more pheromone on the way back from the safe feeder relative to the risky one (GLMM post-hoc with estimated means, estimate=1.28, SE=0.14, z=9.149, p<0.0001).

Experiment 2 – Risk preference between options of different absolute value

Considering pheromone deposited towards the drop, we found an effect of the molarity (figure S4B, GLMM Analysis of Deviance, Chi square=7.489, DF=2, p=0.024). However, post-hoc analysis revealed no difference between any of the molarities: the differences were probably so small that the Bonferroni correction in the post-hoc analysis brought them above significance.

Considering the pheromone deposited back to the nest, we found an effect of molarity (GLMM Analysis of Deviance, Chi square=133.424, DF=1, p<0.0001), an effect of visit (GLMM, Chi square=10.249, DF=1, p=0.001), and an effect of their interaction (GLMM, Chi square=11.339, DF=2, p=0.003). Ants deposited less pheromone for the 0.1M drop in comparison to the 0.55M drop (figure S4E, GLMM post-hoc with estimated means, estimate=−2.683, SE=0.17, z=−15.742, p<0.0001), less pheromone for the 0.1M in comparison to the 1.5M (GLMM post-hoc with estimated means, estimate=−3.474, SE=0.204, z=−17, p<0.0001) and less for the 0.55M in comparison to the 1.5M (GLMM post-hoc with estimated means, estimate=−0.79, SE=0.19, z=−4.144, p=0.0001). Overall the ants deposited more pheromone on the way back from the safe feeder relative to the risky one (GLMM post-hoc with estimated means, estimate=0.947, SE=0.14, z=6.341, p<0.0001).

Experiment 3 – Risk preference between psychophysically-balanced options

Considering pheromone depositions towards the feeder, we found an effect of molarity (GLMM, Chi square=16.133, DF=2, p=0.0003). Ants deposited more pheromone when going towards the 0.3M drop in comparison to the 0.9M drop (figure S4C, GLMM post-hoc with estimated means, estimate=10.444, SE=1.751, z=3.769, p=0.0007), while we found no difference between 0.1M and 0.3M (GLMM post-hoc with estimated means, estimate=0.477, SE=0.174, z=−2.032, p=0.169) and between 0.1M and 0.9M (GLMM post-hoc with estimated means, estimate=4.981, SE=3.452, z=2.317, p=0.082). Overall, ants deposited more pheromone for the safe feeder (GLMM post-hoc with estimated means, estimate=4.679, SE=1.751, z=4.124, p=0.0001)

Considering pheromone deposition back to the nest, we found an effect of molarity (GLMM, Chi square=12.713, DF=2, p=0.0017) and an effect of the interaction between visit number and molarity (GLMM, Chi square=9.447, DF=2, p=0.0088). Ants deposited less pheromone for the 0.3M in comparison to the 0.9M (GLMM post-hoc with estimated means, estimate=−0.515, SE=0.144, z=−3.569, p=0.0014). Overall the ants deposited the same amount of pheromone on the way back from the safe feeder relative to the risky one (GLMM post-hoc with estimated means, estimate=−0.252, SE=0.211, z=−1.199, p=0.922).

Experiment 4 – Risk preference with a psychophysically higher-valued risky alternative.

Considering pheromone depositions towards the feeder, we found no effect of molarity (GLMM, Chi square=2.621, DF=2, p=0.2696) nor visit number (GLMM, Chi square=0.038, DF=1, p=0.8455), nor the interaction between the two (GLMM, Chi square=0.691, DF=2, p=0.708), meaning that they deposited the same amount of pheromone on the way towards all drops.

Considering pheromone deposition back to the nest, we found an effect of molarity (GLMM, Chi square=121.1146, DF=2, p<0.0001). Ants deposited less pheromone when returning from the 0.25M drop in comparison to the 0.5M one (GLMM post-hoc with estimated means, estimate=−1.078, SE=0.191, z=−5.657, p<0.0001), less for the 0.25M in comparison to the 2.0M (GLMM post-hoc with estimated means, estimate=−2.531, SE=0.249, z=−10.175, p<0.0001) and less for the 0.5M in comparison to the 2.0M (GLMM post-hoc with estimated means, estimate=−1.453, SE=0.187, z=−7.768, p<0.0001). Overall the ants deposited the same amount of pheromone on the way back from the safe feeder relative to the risky one (GLMM post-hoc with estimated means, estimate=−0.187, SE=0.142, z=−1.319, p=0.7492).

Experiment 5 – Risk preference with a*n absolutely* higher-valued risky alternative.

Considering pheromone depositions towards the feeder, we found no effect of molarity (GLMM, Chi square=0.2882, DF=2, p=0.8658) but an effect of visit number (GLMM, Chi square=7.2851, DF=1, p=0.0069). There was also a slight effect, although not significant, of the interaction between the two (GLMM, Chi square=5.7211, DF=2, p=0.0572). Indeed, the amount of pheromone deposited towards the 0.25M and the 0.5M drops remains the same over the course of the trial, while it increases for the 1.5M drop (GLMM post-hoc trend, slope=0.1364, SE=0.0306, z=4.455, p<0.0001)

Considering pheromone deposition back to the nest, we found an effect of molarity (GLMM, Chi square=38.663, DF=2, p<0.0001) and an effect of the interaction between molarity and visit number (GLMM, Chi square=8.191, DF=2, p=0.0166). As expected, ants deposited the same amount of pheromone when returning from the 0.25M drop, regardless of what odour it had (and so to what feeder it was associated. GLMM post-hoc with estimated means, estimate=−0361, SE=0.184, z=−1.956, p=0.202). Instead, ants deposited less pheromone when returning from the 0.25M drop in comparison to the 1.5M one, both when the former was associated with the risky feeder (GLMM post-hoc with estimated means, estimate=−1.186, SE=0.193, z=−6.151, p<0.0001) and to the safe one (GLMM post-hoc with estimated means, estimate=−0.825, SE=0.151, z=−5.466, p<0.0001). Overall the ants deposited the same amount of pheromone on the way back from the safe feeder relative to the risky one (GLMM post-hoc with estimated means, estimate=−0.232, SE=0.138, z=−1.68, p=0.372).

Discussion

Does our understanding of individual behaviour in a risk-choice situation help explain the risk indifference of ants at a colony level (Hübner and Czaczkes 2017)? Pheromone deposition rates of individual foragers vary hugely between individuals, even when presented with identical food sources. This is to be expected, given the fact that individual variability may aid collective decisions (Dussutour et al. 2009; O'Shea-Wheller et al. 2017). However, the appropriate measure of pheromone for colony-level decisions is total pheromone deposited. Examining the mean deposition rates for both feeders in experiment 1, we see that ants, on average, deposited more pheromone to the safe feeder (5.5 dots per ant) than the risky feeder (3.9 dots per ants; for the full data see ESM1). In Hübner & Czaczkes (2017) each ant made only one or two visits to the feeder, but even when considering only the first two visits, ants made more pheromone depositions to the safe (1.5 dots per ant) than to the risky (0.89 dots per ant) feeder. The finding of risk neutrality at the colony level is thus still a puzzle. However, the two experiments are not directly comparable. Firstly, in the current experiment pheromone was removed from the trail every visit. Pheromone presence is known to reduce further pheromone deposition (Czaczkes et al. 2013), perhaps damping out the differences between the two feeders. Secondly, the presence of odours on a path affects pheromone deposition: while pheromone deposition on odourless paths is usually higher on the nestward journey (Beckers et al. 1993; Czaczkes et al. 2013, 2016; Czaczkes and Heinze 2015), pheromone deposition is higher on outward journeys on scented paths (This study; Czaczkes et al. 2018b, a). Finally, it should be noted that perception of pheromone, much like perception of quality, is also not linear (von Thienen et al. 2014), thus emphasising initial differences in pheromone concentration but damping out differences between strong trails. Nevertheless, it seems that colony-level decision-making effectively filters out the ants' individual perceptual constraints (This study; Sasaki and Pratt 2011; Sasaki et al. 2019), but the mechanism used to achieve this is still unknown.

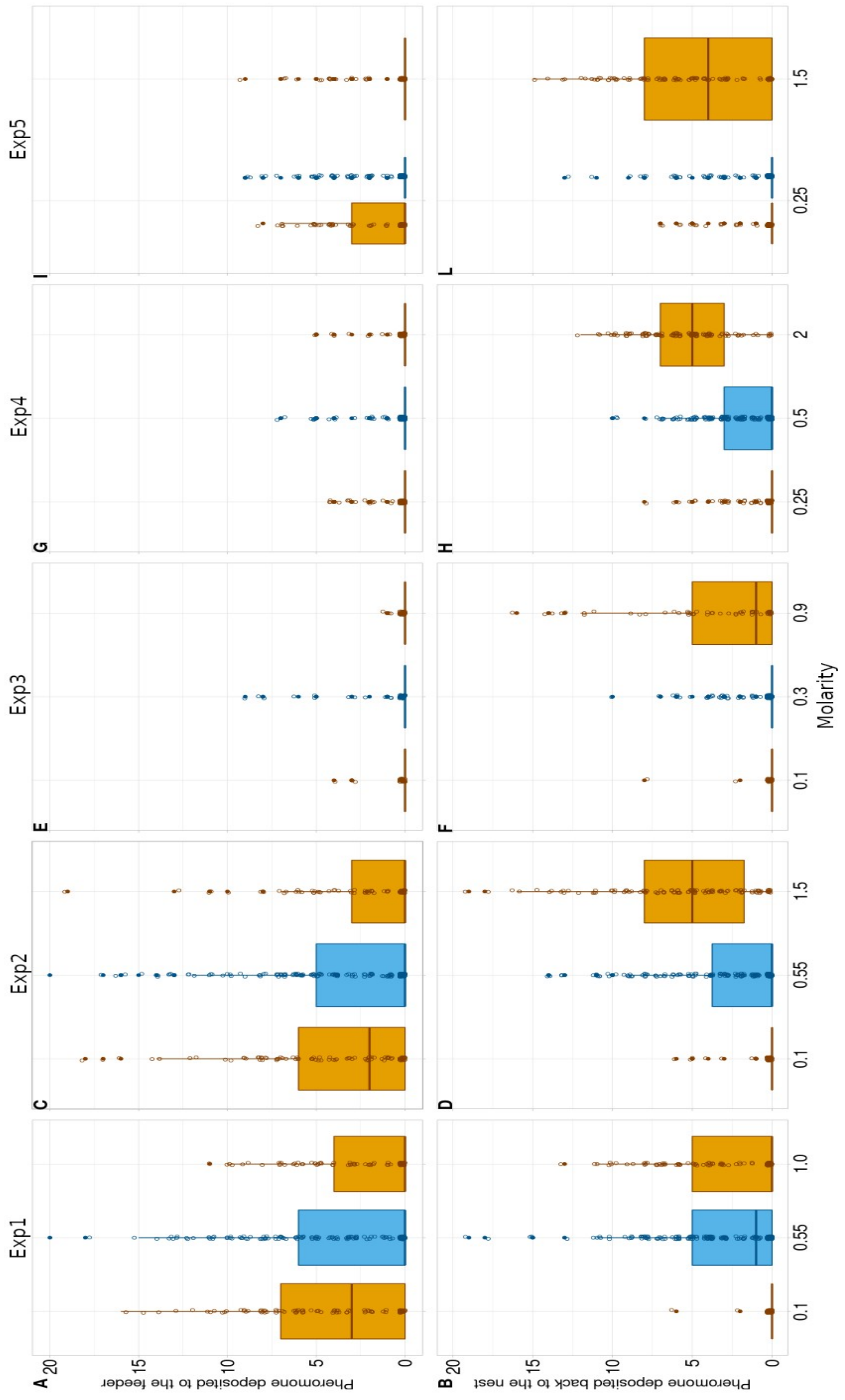

Figure S4 - Frequency of pheromone deposition for the 5 experiments (columns), both for the path to the drop (top row) and the path back to the nest (bottom). In orange, visits in which the risky option is presented. In blue, the safe option. In the box-plots, the line a the centre of the box represents the median value; the coloured area contains al values of the second and third quartile, the lines on top and below the boxes represent the first and fourth quartiles.

4 References

Beckers R, Deneubourg JL, Goss S (1993) Modulation of trail laying in the antLasius niger (Hymenoptera: Formicidae) and its role in the collective selection of a food source. J Insect Behav 6:751–759. https://doi.org/10.1007/BF01201674

Czaczkes TJ, Brandstetter B, di Stefano I, Heinze J (2018a) Greater effort increases perceived value in an invertebrate. J Comp Psychol 132:200–209. https://doi.org/10.1037/com0000109

Czaczkes TJ, Grüter C, Ratnieks FLW (2013) Negative feedback in ants: crowding results in less trail pheromone deposition. Journal of The Royal Society Interface 10:20121009. https://doi.org/10.1098/rsif.2012.1009

Czaczkes TJ, Heinze J (2015) Ants adjust their pheromone deposition to a changing environment and their probability of making errors. Proc Biol Sci 282:. https://doi.org/10.1098/rspb.2015.0679

Czaczkes TJ, Koch A, Fröber K, Dreisbach G (2018b) Voluntary switching in an invertebrate: The effect of cue and reward change. Journal of Experimental Psychology: Animal Learning and Cognition 44:247–257. https://doi.org/10.1037/xan0000171

Czaczkes TJ, Weichselgartner T, Bernadou A, Heinze J (2016) The Effect of Trail Pheromone and Path Confinement on Learning of Complex Routes in the Ant Lasius niger. PLOS ONE 11:e0149720. https://doi.org/10.1371/journal.pone.0149720

Dussutour A, Beekman M, Nicolis SC, Meyer B (2009) Noise improves collective decision-making by ants in dynamic environments. Proc Biol Sci 276:4353–4361. https://doi.org/10.1098/rspb.2009.1235

Hübner C, Czaczkes TJ (2017) Risk preference during collective decision making: ant colonies make risk-indifferent collective choices. Animal Behaviour 132:21–28. https://doi.org/10.1016/j.anbehav.2017.08.003

O'Shea-Wheller TA, Masuda N, Sendova-Franks AB, Franks NR (2017) Variability in individual assessment behaviour and its implications for collective decision-making. Proc R Soc B 284:20162237. https://doi.org/10.1098/rspb.2016.2237

Sasaki T, Pratt SC (2011) Emergence of group rationality from irrational individuals. Behav Ecol 22:276–281. https://doi.org/10.1093/beheco/arq198

Sasaki T, Stott B, Pratt SC (2019) Rational time investment during collective decision making in Temnothorax ants. Biology Letters 15:20190542. https://doi.org/10.1098/rsbl.2019.0542

von Thienen W, Metzler D, Choe D-H, Witte V (2014) Pheromone communication in ants: a detailed analysis of concentration-dependent decisions in three species. Behav Ecol Sociobiol 68:1611–1627. https://doi.org/10.1007/s00265-014-1770-3
