# Supplementary material for: Irrational risk aversion in an ant
Source: Anim Cogn. 2021 May 3;24(6):1237–45. doi: 10.1007/s10071-021-01516-1 (PMC8492575; doi:10.1007/s10071-021-01516-1)
Supplement: Supplementary file 2 — Supplementary file2 (HTML 4241 KB) [file 10071_2021_1516_MOESM2_ESM.html]

Supplemental material 2


# Supplemental material 2

### Data Analysis

Abstract

This supplement provides the entire R script and output of the statistical analysis we performed and figures produced, in their original form. It is presented in the spirit of open and transparent science, but has not been carefully curated.

# Column descriptions

| column\_name | description |
| --- | --- |
| date | Testing date |
| colony | Colony number |
| antN | Ant number |
| condition | Experimental condition |
| AritDiff | Aritmetical average difference between safe and risky |
| GeomDiff | Geometrical average difference between safe and risky |
| antID | Ant individual ID |
| safescent | Scent of the safe feeder |
| riskscent | Scent of the risky feeder |
| firstrisk | Which of the risky alternatives is presented first |
| firstfeed | Which of the two feeders is presented first |
| visit | Visit number |
| type | Training or testing visit |
| mol | molarity of the drop in the current visit |
| lastmol | molarity of the drop in the previous visit |
| lastfeedmol | molarity of the drop in the previous visit of the same feeder |
| scent | scent of the current visit |
| feed | feeder of the current visit |
| phergo | pheromone deposited on the way to the drop |
| pherbk | pheromone deposited on the way back to the nest |
| safeside | side of the safe smell in the Y maze test |
| firstchoice | initial choice side |
| endchoice | final choice side |
| firstchoicesafe | initial choice binomial data (safe is 1) |
| endchoicesafe | final choice binomial data (safe is 1) |

# Data analysis

first I load packages

```
library(lme4)
library(DHARMa)
library(car)
library(emmeans)
library(reshape2)
library(ggplot2)
library(knitr)
library(pscl)

set.seed(123)#set seed for replicability in random simulations
```

## Binomial Choice

### Preliminary questions

#### initial vs. final

first, I want to know if initial and final choice differ

##### exp 1

```
fsdiff<-melt(exp1, measure.vars = c("firstchoicesafe","endchoicesafe"))

mdiff<-glmer(value~variable+(1|colony/antID),data=fsdiff,family=binomial)
```

```
## boundary (singular) fit: see ?isSingular
```

```
Anova(mdiff)
```

```
## Analysis of Deviance Table (Type II Wald chisquare tests)
## 
## Response: value
##           Chisq Df Pr(>Chisq)
## variable 0.5111  1     0.4747
```

```
e<-emmeans(mdiff, ~variable, type="response")
pairs(e)
```

```
##  contrast                        odds.ratio    SE  df z.ratio p.value
##  firstchoicesafe / endchoicesafe      0.814 0.234 Inf -0.715  0.4747 
## 
## Tests are performed on the log odds ratio scale
```

there is no difference between initial and final choice, I will now on only use the initial for further analysis

##### exp 2

```
fsdiff<-melt(exp2, measure.vars = c("firstchoicesafe","endchoicesafe"))

mdiff<-glmer(value~variable+(1|colony/antID),data=fsdiff,family=binomial)
Anova(mdiff)
```

```
## Analysis of Deviance Table (Type II Wald chisquare tests)
## 
## Response: value
##           Chisq Df Pr(>Chisq)
## variable 0.2903  1       0.59
```

```
e<-emmeans(mdiff, ~variable, type="response")
pairs(e)
```

```
##  contrast                        odds.ratio    SE  df z.ratio p.value
##  firstchoicesafe / endchoicesafe        1.1 0.195 Inf 0.539   0.5900 
## 
## Tests are performed on the log odds ratio scale
```

there is no difference between initial and final choice, I will now on only use the initial for further analysis

##### exp 3

```
fsdiff<-melt(exp3, measure.vars = c("firstchoicesafe","endchoicesafe"))

mdiff<-glmer(value~variable+(1|colony/antID),data=fsdiff,family=binomial)
```

```
## boundary (singular) fit: see ?isSingular
```

```
Anova(mdiff)
```

```
## Analysis of Deviance Table (Type II Wald chisquare tests)
## 
## Response: value
##           Chisq Df Pr(>Chisq)
## variable 0.1981  1     0.6563
```

```
e<-emmeans(mdiff, ~variable, type="response")
pairs(e)
```

```
##  contrast                        odds.ratio    SE  df z.ratio p.value
##  firstchoicesafe / endchoicesafe        1.1 0.243 Inf 0.445   0.6563 
## 
## Tests are performed on the log odds ratio scale
```

there is no difference between initial and final choice, I will now on only use the initial for further analysis

##### exp 4

```
fsdiff<-melt(exp4, measure.vars = c("firstchoicesafe","endchoicesafe"))

mdiff<-glmer(value~variable+(1|colony/antID),data=fsdiff,family=binomial)
```

```
## boundary (singular) fit: see ?isSingular
```

```
Anova(mdiff)
```

```
## Analysis of Deviance Table (Type II Wald chisquare tests)
## 
## Response: value
##           Chisq Df Pr(>Chisq)
## variable 0.1093  1     0.7409
```

```
e<-emmeans(mdiff, ~variable, type="response")
pairs(e)
```

```
##  contrast                        odds.ratio    SE  df z.ratio p.value
##  firstchoicesafe / endchoicesafe       1.08 0.236 Inf 0.331   0.7409 
## 
## Tests are performed on the log odds ratio scale
```

there is no difference between initial and final choice, I will now on only use the initial for further analysis

##### exp 5

```
fsdiff<-melt(exp5, measure.vars = c("firstchoicesafe","endchoicesafe"))

mdiff<-glmer(value~variable+(1|colony/antID),data=fsdiff,family=binomial)
Anova(mdiff)
```

```
## Analysis of Deviance Table (Type II Wald chisquare tests)
## 
## Response: value
##           Chisq Df Pr(>Chisq)
## variable 0.0761  1     0.7827
```

```
e<-emmeans(mdiff, ~variable, type="response")
pairs(e)
```

```
##  contrast                        odds.ratio    SE  df z.ratio p.value
##  firstchoicesafe / endchoicesafe       1.08 0.298 Inf 0.276   0.7827 
## 
## Tests are performed on the log odds ratio scale
```

there is no difference between initial and final choice, I will now on only use the initial for further analysis

#### visits n.

now, I want to know if the visits differ from one another

##### exp 1

```
exp1$visit<-as.numeric(exp1$visit)
mvisdiff<-glmer(firstchoicesafe~visit+(1|colony/antID),data=exp1,family=binomial,
                glmerControl(optimizer="bobyqa", optCtrl = list(maxfun = 1000000000)))
```

```
## Warning in checkConv(attr(opt, "derivs"), opt$par, ctrl = control$checkConv, :
## Model failed to converge with max|grad| = 0.0157628 (tol = 0.002, component 1)
```

```
mvisdiff<-glmer(firstchoicesafe~visit+(1|antID),data=exp1,family=binomial,
                glmerControl(optimizer="bobyqa", optCtrl = list(maxfun = 1000000000)))
Anova(mvisdiff)
```

```
## Analysis of Deviance Table (Type II Wald chisquare tests)
## 
## Response: firstchoicesafe
##        Chisq Df Pr(>Chisq)   
## visit 9.6688  1   0.001874 **
## ---
## Signif. codes:  0 '***' 0.001 '**' 0.01 '*' 0.05 '.' 0.1 ' ' 1
```

```
summary(mvisdiff)
```

```
## Generalized linear mixed model fit by maximum likelihood (Laplace
##   Approximation) [glmerMod]
##  Family: binomial  ( logit )
## Formula: firstchoicesafe ~ visit + (1 | antID)
##    Data: exp1
## Control: glmerControl(optimizer = "bobyqa", optCtrl = list(maxfun = 1e+09))
## 
##      AIC      BIC   logLik deviance df.resid 
##    196.7    206.4    -95.3    190.7      189 
## 
## Scaled residuals: 
##     Min      1Q  Median      3Q     Max 
## -2.8804  0.3022  0.3472  0.5081  0.8520 
## 
## Random effects:
##  Groups Name        Variance Std.Dev.
##  antID  (Intercept) 0.3172   0.5632  
## Number of obs: 192, groups:  antID, 64
## 
## Fixed effects:
##             Estimate Std. Error z value Pr(>|z|)    
## (Intercept)   9.0752     2.5511   3.557 0.000375 ***
## visit        -0.7616     0.2449  -3.109 0.001874 ** 
## ---
## Signif. codes:  0 '***' 0.001 '**' 0.01 '*' 0.05 '.' 0.1 ' ' 1
## 
## Correlation of Fixed Effects:
##       (Intr)
## visit -0.996
```

the percentage of ants choosing safe decreases with successive visits. this means that more and more ants after not finding the sugar drop start doing a random search. I will from now on only observe the first visit, being it a clearer indication of choice

##### exp 2

```
mvisdiff<-glmer(firstchoicesafe~visit+(1|colony/antID),data=exp2,family=binomial,
                glmerControl(optimizer="bobyqa", optCtrl = list(maxfun = 100000)))
Anova(mvisdiff)
```

```
## Analysis of Deviance Table (Type II Wald chisquare tests)
## 
## Response: firstchoicesafe
##       Chisq Df Pr(>Chisq)  
## visit 5.885  1    0.01527 *
## ---
## Signif. codes:  0 '***' 0.001 '**' 0.01 '*' 0.05 '.' 0.1 ' ' 1
```

```
summary(mvisdiff)
```

```
## Generalized linear mixed model fit by maximum likelihood (Laplace
##   Approximation) [glmerMod]
##  Family: binomial  ( logit )
## Formula: firstchoicesafe ~ visit + (1 | colony/antID)
##    Data: exp2
## Control: glmerControl(optimizer = "bobyqa", optCtrl = list(maxfun = 1e+05))
## 
##      AIC      BIC   logLik deviance df.resid 
##    415.4    430.4   -203.7    407.4      316 
## 
## Scaled residuals: 
##     Min      1Q  Median      3Q     Max 
## -1.8473 -1.1416  0.6020  0.7331  1.0362 
## 
## Random effects:
##  Groups       Name        Variance Std.Dev.
##  antID:colony (Intercept) 0.14277  0.3778  
##  colony       (Intercept) 0.03706  0.1925  
## Number of obs: 320, groups:  antID:colony, 64; colony, 8
## 
## Fixed effects:
##             Estimate Std. Error z value Pr(>|z|)   
## (Intercept)  2.96566    0.97582   3.039  0.00237 **
## visit       -0.20978    0.08647  -2.426  0.01527 * 
## ---
## Signif. codes:  0 '***' 0.001 '**' 0.01 '*' 0.05 '.' 0.1 ' ' 1
## 
## Correlation of Fixed Effects:
##       (Intr)
## visit -0.988
```

the percentage of ants choosing safe decreases with successive visits. this means that more and more ants after not finding the sugar drop start doing a random search. I will from now on only observe the first visit, being it a clearer indication of choice

##### exp 3

```
mvisdiff<-glmer(firstchoicesafe~visit+(1|colony/antID),data=exp3,family=binomial,
                glmerControl(optimizer="bobyqa", optCtrl = list(maxfun = 100000)))
```

```
## boundary (singular) fit: see ?isSingular
```

```
Anova(mvisdiff)
```

```
## Analysis of Deviance Table (Type II Wald chisquare tests)
## 
## Response: firstchoicesafe
##        Chisq Df Pr(>Chisq)
## visit 0.5281  1     0.4674
```

```
summary(mvisdiff)
```

```
## Generalized linear mixed model fit by maximum likelihood (Laplace
##   Approximation) [glmerMod]
##  Family: binomial  ( logit )
## Formula: firstchoicesafe ~ visit + (1 | colony/antID)
##    Data: exp3
## Control: glmerControl(optimizer = "bobyqa", optCtrl = list(maxfun = 1e+05))
## 
##      AIC      BIC   logLik deviance df.resid 
##    282.4    295.6   -137.2    274.4      196 
## 
## Scaled residuals: 
##     Min      1Q  Median      3Q     Max 
## -1.1864 -0.9476  0.6974  0.9092  1.2280 
## 
## Random effects:
##  Groups       Name        Variance Std.Dev.
##  antID:colony (Intercept) 0.3117   0.5583  
##  colony       (Intercept) 0.0000   0.0000  
## Number of obs: 200, groups:  antID:colony, 40; colony, 10
## 
## Fixed effects:
##             Estimate Std. Error z value Pr(>|z|)
## (Intercept)  0.94152    1.16050   0.811    0.417
## visit       -0.07576    0.10425  -0.727    0.467
## 
## Correlation of Fixed Effects:
##       (Intr)
## visit -0.989
## convergence code: 0
## boundary (singular) fit: see ?isSingular
```

there is no difference between visits. I will use only first for consistency, but I expect random choice. in this case, it would be clear why there is no decrease: if the choice is already random there is no room for reverting to random choice with subsequent visits.

##### exp 4

```
mvisdiff<-glmer(firstchoicesafe~visit+(1|colony/antID),data=exp4,family=binomial,
                glmerControl(optimizer="bobyqa", optCtrl = list(maxfun = 100000)))
```

```
## boundary (singular) fit: see ?isSingular
```

```
Anova(mvisdiff)
```

```
## Analysis of Deviance Table (Type II Wald chisquare tests)
## 
## Response: firstchoicesafe
##        Chisq Df Pr(>Chisq)
## visit 0.7901  1     0.3741
```

```
summary(mvisdiff)
```

```
## Generalized linear mixed model fit by maximum likelihood (Laplace
##   Approximation) [glmerMod]
##  Family: binomial  ( logit )
## Formula: firstchoicesafe ~ visit + (1 | colony/antID)
##    Data: exp4
## Control: glmerControl(optimizer = "bobyqa", optCtrl = list(maxfun = 1e+05))
## 
##      AIC      BIC   logLik deviance df.resid 
##    273.1    286.2   -132.6    265.1      188 
## 
## Scaled residuals: 
##     Min      1Q  Median      3Q     Max 
## -1.0660 -0.9557 -0.8569  1.0158  1.1330 
## 
## Random effects:
##  Groups       Name        Variance Std.Dev.
##  antID:colony (Intercept) 0.06197  0.2489  
##  colony       (Intercept) 0.00000  0.0000  
## Number of obs: 192, groups:  antID:colony, 64; colony, 5
## 
## Fixed effects:
##             Estimate Std. Error z value Pr(>|z|)
## (Intercept)  -1.6555     1.7977  -0.921    0.357
## visit         0.1592     0.1791   0.889    0.374
## 
## Correlation of Fixed Effects:
##       (Intr)
## visit -0.997
## convergence code: 0
## boundary (singular) fit: see ?isSingular
```

there seem to be no decrease also here

##### exp 5

```
mvisdiff<-glmer(firstchoicesafe~visit+(1|colony/antID),data=exp5,family=binomial,
                glmerControl(optimizer="bobyqa", optCtrl = list(maxfun = 100000)))
```

```
## Warning in checkConv(attr(opt, "derivs"), opt$par, ctrl = control$checkConv, :
## Model failed to converge with max|grad| = 0.0341977 (tol = 0.002, component 1)
```

```
mvisdiff<-glmer(firstchoicesafe~visit+(1|antID),data=exp5,family=binomial,
                glmerControl(optimizer="bobyqa", optCtrl = list(maxfun = 100000)))
Anova(mvisdiff)
```

```
## Analysis of Deviance Table (Type II Wald chisquare tests)
## 
## Response: firstchoicesafe
##        Chisq Df Pr(>Chisq)
## visit 0.7255  1     0.3944
```

```
summary(mvisdiff)
```

```
## Generalized linear mixed model fit by maximum likelihood (Laplace
##   Approximation) [glmerMod]
##  Family: binomial  ( logit )
## Formula: firstchoicesafe ~ visit + (1 | antID)
##    Data: exp5
## Control: glmerControl(optimizer = "bobyqa", optCtrl = list(maxfun = 1e+05))
## 
##      AIC      BIC   logLik deviance df.resid 
##    227.7    237.5   -110.9    221.7      189 
## 
## Scaled residuals: 
##     Min      1Q  Median      3Q     Max 
## -0.8817 -0.5553 -0.4488  0.9518  1.8008 
## 
## Random effects:
##  Groups Name        Variance Std.Dev.
##  antID  (Intercept) 0.8803   0.9382  
## Number of obs: 192, groups:  antID, 64
## 
## Fixed effects:
##             Estimate Std. Error z value Pr(>|z|)
## (Intercept)  -2.9907     2.1850  -1.369    0.171
## visit         0.1834     0.2153   0.852    0.394
## 
## Correlation of Fixed Effects:
##       (Intr)
## visit -0.994
```

there seem to be no decrease also here

### modeling

now to the actual model. I drop antID because I kept only one observation for each ant

#### Exp 1

```
exp1sing<-subset(exp1,exp1$visit==9)

mExp1<-glmer(firstchoicesafe~firstfeed*firstrisk+(1|colony),data=exp1sing,family=binomial,
             glmerControl(optimizer="bobyqa", optCtrl = list(maxfun = 100000)))
```

```
## boundary (singular) fit: see ?isSingular
```

```
simres<-simulateResiduals(mExp1) #standard seed for random values is 123
plot(simres, asFactor=T)
```

model is good here

```
Anova(mExp1)
```

```
## Analysis of Deviance Table (Type II Wald chisquare tests)
## 
## Response: firstchoicesafe
##                      Chisq Df Pr(>Chisq)
## firstfeed           0.7092  1     0.3997
## firstrisk           0.0000  1     1.0000
## firstfeed:firstrisk 0.0000  1     1.0000
```

no effect of any of the factors. will just test overall preference

```
meanobj <- emmeans(mExp1,~1, type="response")
(test(meanobj))
```

```
##  1        prob     SE  df z.ratio p.value
##  overall 0.911 0.0367 Inf 5.142   <.0001 
## 
## Results are averaged over the levels of: firstfeed, firstrisk 
## Tests are performed on the logit scale
```

ants prefer the safe **91%**

#### Exp 2

```
exp2sing<-subset(exp2,exp2$visit==9)

mExp2<-glmer(firstchoicesafe~firstfeed*firstrisk+(1|colony),data=exp2sing,family=binomial,
             glmerControl(optimizer="bobyqa", optCtrl = list(maxfun = 100000)))

simres<-simulateResiduals(mExp2) #standard seed for random values is 123
plot(simres, asFactor=T)
```

good model also here

```
Anova(mExp2)
```

```
## Analysis of Deviance Table (Type II Wald chisquare tests)
## 
## Response: firstchoicesafe
##                      Chisq Df Pr(>Chisq)
## firstfeed           2.0148  1     0.1558
## firstrisk           0.1969  1     0.6572
## firstfeed:firstrisk 1.8066  1     0.1789
```

still, no effect of factors.

```
meanobj<-emmeans(mExp2,~1, type="response")
(test(meanobj))
```

```
##  1        prob     SE  df z.ratio p.value
##  overall 0.792 0.0678 Inf 3.248   0.0012 
## 
## Results are averaged over the levels of: firstfeed, firstrisk 
## Tests are performed on the logit scale
```

ants prefer the safe **79%**

#### Exp 3

```
exp3sing<-subset(exp3,exp3$visit==9)

mExp3<-glmer(firstchoicesafe~firstfeed*firstrisk+(1|colony),data=exp3sing,family=binomial,
             glmerControl(optimizer="bobyqa", optCtrl = list(maxfun = 100000)))
```

```
## boundary (singular) fit: see ?isSingular
```

```
simres<-simulateResiduals(mExp3) #standard seed for random values is 123
plot(simres, asFactor=T)
```

still, good model

```
Anova(mExp3)
```

```
## Analysis of Deviance Table (Type II Wald chisquare tests)
## 
## Response: firstchoicesafe
##                      Chisq Df Pr(>Chisq)  
## firstfeed           4.4237  1    0.03544 *
## firstrisk           0.0146  1    0.90388  
## firstfeed:firstrisk 0.6679  1    0.41377  
## ---
## Signif. codes:  0 '***' 0.001 '**' 0.01 '*' 0.05 '.' 0.1 ' ' 1
```

there is an effect of the first presented feeder. first of all let’s look at overall percentage

```
meanobj<-emmeans(mExp3,~1, type="response")
(test(meanobj))
```

```
##  1        prob     SE  df z.ratio p.value
##  overall 0.535 0.0864 Inf 0.403   0.6870 
## 
## Results are averaged over the levels of: firstfeed, firstrisk 
## Tests are performed on the logit scale
```

ants prefere the safe **53%**.

```
meanobj<-emmeans(mExp3,~firstfeed, type="response")
```

```
## NOTE: Results may be misleading due to involvement in interactions
```

```
pairs(meanobj)
```

```
##  contrast     odds.ratio   SE  df z.ratio p.value
##  risky / safe      0.216 0.15 Inf -2.207  0.0273 
## 
## Results are averaged over the levels of: firstrisk 
## Tests are performed on the log odds ratio scale
```

```
meanobj
```

```
##  firstfeed  prob    SE  df asymp.LCL asymp.UCL
##  risky     0.348 0.107 Inf     0.175     0.574
##  safe      0.712 0.104 Inf     0.477     0.870
## 
## Results are averaged over the levels of: firstrisk 
## Confidence level used: 0.95 
## Intervals are back-transformed from the logit scale
```

more ants go to the safe when this is presented fist, more ants go to the risky when is presented first. overall is random! probably in front of a random choice they just go for the first experienced.

#### Exp 4

```
exp4sing<-subset(exp4,exp4$visit==9)

mExp4<-glmer(firstchoicesafe~firstfeed*firstrisk+(1|colony),data=exp4sing,family=binomial,
             glmerControl(optimizer="bobyqa", optCtrl = list(maxfun = 100000)))

simres<-simulateResiduals(mExp4) #standard seed for random values is 123
plot(simres, asFactor=T)
```

model is good here

```
Anova(mExp4)
```

```
## Analysis of Deviance Table (Type II Wald chisquare tests)
## 
## Response: firstchoicesafe
##                      Chisq Df Pr(>Chisq)  
## firstfeed           5.4001  1    0.02014 *
## firstrisk           0.4078  1    0.52309  
## firstfeed:firstrisk 0.9631  1    0.32640  
## ---
## Signif. codes:  0 '***' 0.001 '**' 0.01 '*' 0.05 '.' 0.1 ' ' 1
```

there is an effect of the first experienced feeder (or odour), being it the risky or the safe one

```
meanobj <- emmeans(mExp4,~firstfeed, type="response")
```

```
## NOTE: Results may be misleading due to involvement in interactions
```

```
pairs(meanobj, adjust="bonferroni")
```

```
##  contrast     odds.ratio    SE  df z.ratio p.value
##  risky / safe      0.266 0.148 Inf -2.380  0.0173 
## 
## Results are averaged over the levels of: firstrisk 
## Tests are performed on the log odds ratio scale
```

```
meanobj <- emmeans(mExp4,~1, type="response")
test(meanobj)
```

```
##  1        prob    SE  df z.ratio p.value
##  overall 0.427 0.085 Inf -0.844  0.3985 
## 
## Results are averaged over the levels of: firstfeed, firstrisk 
## Tests are performed on the logit scale
```

indeed, ants tend to chose the safe option more when they experience first the risky one. overall, the ants preferred the safe **42.7%** or preferred the risky **57.3%**, a percentage not different from chance level

#### Exp 5

```
exp5sing<-subset(exp5,exp5$visit==9)

mExp5<-glmer(firstchoicesafe~firstfeed*firstrisk+(1|colony),data=exp5sing,family=binomial,
             glmerControl(optimizer="bobyqa", optCtrl = list(maxfun = 100000)))
```

```
## boundary (singular) fit: see ?isSingular
```

```
mExp5<-glm(firstchoicesafe~firstfeed*firstrisk,data=exp5sing,family=binomial)

simres<-simulateResiduals(mExp5) #standard seed for random values is 123
plot(simres, asFactor=T)
```

model is good here

```
Anova(mExp5)
```

```
## Analysis of Deviance Table (Type II tests)
## 
## Response: firstchoicesafe
##                     LR Chisq Df Pr(>Chisq)
## firstfeed             0.1004  1     0.7514
## firstrisk             2.4683  1     0.1162
## firstfeed:firstrisk   1.2439  1     0.2647
```

no effect of any of the factors. will just test overall preference

```
meanobj <- emmeans(mExp5,~1, type="response")
(test(meanobj))
```

```
##  1       prob     SE  df z.ratio p.value
##  overall 0.18 0.0535 Inf -4.182  <.0001 
## 
## Results are averaged over the levels of: firstfeed, firstrisk 
## Tests are performed on the logit scale
```

ants prefer the safe **18%**, or in other words prefer the risky **82%** ### Overall graph

```
exp1sing$blindmol<-NULL
all <- rbind(exp1sing,exp2sing,exp3sing,exp4sing,exp5sing)

library(plyr)
all$condition<-revalue(all$condition, c("BestRisk"="Exp5","BetterRisk"="Exp4","GeomAvrg"="Exp3", "Irrational"="exp2","sameAvg"="Exp1"))
```

```
## The following `from` values were not present in `x`: BestRisk, BetterRisk, GeomAvrg, Irrational, sameAvg
```

```
mTot <- glmer(firstchoicesafe~condition+(1|colony),data=all,family=binomial,
              glmerControl(optimizer="bobyqa", optCtrl = list(maxfun = 100000)))

meanobj<-emmeans(mTot,~condition, type="response")
toplot1<-as.data.frame(meanobj)
(pairs(meanobj))
```

```
##  contrast    odds.ratio     SE  df z.ratio p.value
##  Exp1 / Exp2       3.12  1.735 Inf 2.051   0.2418 
##  Exp1 / Exp3       9.03  5.158 Inf 3.851   0.0011 
##  Exp1 / Exp4      13.26  7.310 Inf 4.690   <.0001 
##  Exp1 / Exp5      39.95 23.207 Inf 6.349   <.0001 
##  Exp2 / Exp3       2.89  1.287 Inf 2.383   0.1199 
##  Exp2 / Exp4       4.25  1.864 Inf 3.292   0.0088 
##  Exp2 / Exp5      12.79  6.083 Inf 5.358   <.0001 
##  Exp3 / Exp4       1.47  0.661 Inf 0.855   0.9132 
##  Exp3 / Exp5       4.43  2.151 Inf 3.060   0.0188 
##  Exp4 / Exp5       3.01  1.226 Inf 2.709   0.0527 
## 
## P value adjustment: tukey method for comparing a family of 5 estimates 
## Tests are performed on the log odds ratio scale
```

```
pdf(file="/home/massimodeagro/Archive_NTFS/Drive/DriveRegensburgLab/Ants/Experients/Submitted_GambleFoodValue/tosubmit/AnimalCognition/ThirdSubmission/Figure_1.pdf")
ggplot(toplot1,aes(x=condition,y=prob))+
  ylab("Proportion of ants choosing safe option")+
  ylim(0,1)+
  scale_x_discrete(name= NULL,
                   limits=c("Exp1","Exp2","Exp3","Exp4","Exp5"),
                   labels=c("Exp1" = "Exp1\nSafe: 0.55\nRisky: 0.1/1.0\nlog diff.: 0.23",
                            "Exp2" = "Exp2\nSafe: 0.55\nRisky: 0.1/1.5\nlog diff.: 0.16",
                            "Exp3" = "Exp3\nSafe: 0.3\nRisky: 0.1/0.9\nlog diff.: 0",
                            "Exp4" = "Exp4\nSafe: 0.5\nRisky: 0.25/2.0\nlog diff.: -0.21",
                            "Exp5" = "Exp5\nSafe: 0.25\nRisky: 0.25/1.5\nlog diff.: -0.36"))+
  theme_light()+
  theme(axis.text.x = element_text(size=12),
        axis.text.y = element_text(size=14),
        axis.title.y = element_text(size=16))+
  geom_label(x=1, y=0, label="n = 64",size=5, aes(fontface=3))+
  geom_label(x=2, y=0, label="n = 64",size=5, aes(fontface=3))+
  geom_label(x=3, y=0, label="n = 40",size=5, aes(fontface=3))+
  geom_label(x=4, y=0, label="n = 64",size=5, aes(fontface=3))+
  geom_label(x=5, y=0, label="n = 64",size=5, aes(fontface=3))+
  geom_hline(yintercept = 0.5,linetype="dotted")+
  geom_point(size=3)+
  geom_errorbar(aes(ymin=prob-SE,ymax=prob+SE))
dev.off()
```

```
## png 
##   2
```

### Comparing the experiment by the difference between alternatives, linear or logarithmic?

```
mTotArit <- glmer(firstchoicesafe~AritDiff+(1|colony),data=all,family=binomial,
              glmerControl(optimizer="bobyqa", optCtrl = list(maxfun = 100000)))
mTotGeom <- glmer(firstchoicesafe~GeomDiff+(1|colony),data=all,family=binomial,
              glmerControl(optimizer="bobyqa", optCtrl = list(maxfun = 100000)))

mTotArit <- glm(firstchoicesafe~AritDiff,data=all,family=binomial)
mTotGeom <- glm(firstchoicesafe~GeomDiff,data=all,family=binomial)

library(nonnest2)
```

```
## This is nonnest2 0.5-5.
## nonnest2 has not been tested with all combinations of model classes.
```

```
vuongtest(mTotArit, mTotGeom)
```

```
## Warning in imhof(n * omega.hat.2, lamstar^2): Note that Qq + abserr is positive.
```

```
## 
## Model 1 
##  Class: glm 
##  Call: glm(formula = firstchoicesafe ~ AritDiff, family = binomial, ...
## 
## Model 2 
##  Class: glm 
##  Call: glm(formula = firstchoicesafe ~ GeomDiff, family = binomial, ...
## 
## Variance test 
##   H0: Model 1 and Model 2 are indistinguishable 
##   H1: Model 1 and Model 2 are distinguishable 
##     w2 = 0.034,   p = <2e-16
## 
## Non-nested likelihood ratio test 
##   H0: Model fits are equal for the focal population 
##   H1A: Model 1 fits better than Model 2 
##     z = -2.003,   p = 0.977
##   H1B: Model 2 fits better than Model 1 
##     z = -2.003,   p = 0.02257
```

model 2, based on the geometrical average, is a significantly better fit than model 1

## Pheromone deposition

I will look at the pheromone deposited on the way to the drop and back to the nest for each experiment across visits.

### Exp1

#### To the drop

```
 exp1$visit<-as.numeric(exp1$visit)

mpExp1<-glmer(phergo~visit*mol+(1|colony/antID),data=exp1,family=poisson,
              glmerControl(optimizer="bobyqa", optCtrl = list(maxfun = 100000)))
```

```
## boundary (singular) fit: see ?isSingular
```

```
simres<-simulateResiduals(mpExp1) #standard seed for random values is 123
plot(simres, asFactor=T)
```

the model is zero inflated. let’s remodel

```
mpExp1 <- zeroinfl(phergo ~ visit*mol + 1 | colony/antID, data = exp1)
```

```
## Error in optim(fn = loglikfun, gr = gradfun, par = c(start$count, start$zero, : valore non finito fornito da optim
```

does not work. I will remove colony from random effect.

```
mpExp1 <- zeroinfl(phergo ~ visit*mol + 1 | antID, data = exp1)

Anova(mpExp1)
```

```
## Analysis of Deviance Table (Type II tests)
## 
## Response: phergo
##           Df   Chisq Pr(>Chisq)    
## visit      1  1.7587  0.1847904    
## mol        2 12.9922  0.0015093 ** 
## visit:mol  2 14.4691  0.0007212 ***
## ---
## Signif. codes:  0 '***' 0.001 '**' 0.01 '*' 0.05 '.' 0.1 ' ' 1
```

```
meanobj<-emmeans(mpExp1,~visit*mol,type="response")
contrast(meanobj,list(mol0.1vs0.55=c(1,-1,0),
                      mol0.1vs1.0=c(1,0,-1),
                      mol0.55vs1.0=c(0,1,-1),
                      SafeVsRisky=c(-0.5,1,-0.5)),
         adjust="bonferroni")
```

```
##  contrast     estimate    SE  df z.ratio p.value
##  mol0.1vs0.55   -0.339 0.224 Inf -1.508  0.5258 
##  mol0.1vs1.0     0.319 0.243 Inf  1.311  0.7600 
##  mol0.55vs1.0    0.657 0.227 Inf  2.891  0.0154 
##  SafeVsRisky     0.498 0.190 Inf  2.616  0.0356 
## 
## Results are averaged over the levels of: antID 
## P value adjustment: bonferroni method for 4 tests
```

```
exp1train<-subset(exp1,exp1$visit<9) #just remove tests
exp1train <- droplevels(exp1train)
```

#### Back to the nest

```
mpExp1<-glmer(pherbk~visit*mol+(1|colony/antID),data=exp1,family=poisson,
              glmerControl(optimizer="bobyqa", optCtrl = list(maxfun = 100000)))
simres<-simulateResiduals(mpExp1) #standard seed for random values is 123
plot(simres, asFactor=T)
```

the model is zero inflated. let’s remodel

```
mpExp1 <- zeroinfl(pherbk ~ visit*mol + 1 | colony/antID, data = exp1)
```

```
## Error in optim(fn = loglikfun, gr = gradfun, par = c(start$count, start$zero, : valore non finito fornito da optim
```

does not work. I will remove colony from random effect.

```
mpExp1 <- zeroinfl(pherbk ~ visit*mol + 1 | antID, data = exp1)

Anova(mpExp1)
```

```
## Analysis of Deviance Table (Type II tests)
## 
## Response: pherbk
##           Df   Chisq Pr(>Chisq)    
## visit      1  5.1128    0.02375 *  
## mol        2 85.9724    < 2e-16 ***
## visit:mol  2  3.9549    0.13842    
## ---
## Signif. codes:  0 '***' 0.001 '**' 0.01 '*' 0.05 '.' 0.1 ' ' 1
```

```
meanobj<-emmeans(mpExp1,~visit*mol,type="response")
contrast(meanobj,list(mol0.1vs0.55=c(1,-1,0),
                      mol0.1vs1.0=c(1,0,-1),
                      mol0.55vs1.0=c(0,1,-1),
                      SafeVsRisky=c(-0.5,1,-0.5)),
         adjust="bonferroni")
```

```
##  contrast     estimate    SE  df z.ratio p.value
##  mol0.1vs0.55   -2.670 0.154 Inf -17.352 <.0001 
##  mol0.1vs1.0    -2.780 0.194 Inf -14.308 <.0001 
##  mol0.55vs1.0   -0.111 0.185 Inf  -0.597 1.0000 
##  SafeVsRisky     1.280 0.140 Inf   9.149 <.0001 
## 
## Results are averaged over the levels of: antID 
## P value adjustment: bonferroni method for 4 tests
```

### Exp2

#### To the drop

```
exp2$visit<-as.numeric(exp2$visit)

mpExp2<-glmer(phergo~visit*mol+(1|colony/antID),data=exp2,family=poisson,
              glmerControl(optimizer="bobyqa", optCtrl = list(maxfun = 100000)))
simres<-simulateResiduals(mpExp2) #standard seed for random values is 123
plot(simres, asFactor=T)
```

the model is zero inflated. let’s remodel

```
mpExp2 <- zeroinfl(phergo ~ visit*mol + 1 | colony/antID, data = exp2)
```

```
## Error in optim(fn = loglikfun, gr = gradfun, par = c(start$count, start$zero, : valore non finito fornito da optim
```

does not work. I will remove colony from random effect.

```
exp2$mol<-as.factor(exp2$mol)
mpExp2 <- zeroinfl(phergo ~ visit*mol + 1 | antID, data = exp2)

Anova(mpExp2)
```

```
## Analysis of Deviance Table (Type II tests)
## 
## Response: phergo
##           Df  Chisq Pr(>Chisq)  
## visit      1 0.2799    0.59679  
## mol        2 7.4888    0.02365 *
## visit:mol  2 1.6650    0.43496  
## ---
## Signif. codes:  0 '***' 0.001 '**' 0.01 '*' 0.05 '.' 0.1 ' ' 1
```

```
meanobj<-emmeans(mpExp2,~visit*mol,type="response")
contrast(meanobj,list(mol0.1vs0.55=c(1,-1,0),
                      mol0.1vs1.5=c(1,0,-1),
                      mol0.55vs1.5=c(0,1,-1),
                      SafeVsRisky=c(-0.5,1,-0.5)),
         adjust="bonferroni")
```

```
##  contrast     estimate    SE  df z.ratio p.value
##  mol0.1vs0.55   -0.174 0.229 Inf -0.760  1.0000 
##  mol0.1vs1.5     0.323 0.265 Inf  1.217  0.8944 
##  mol0.55vs1.5    0.497 0.233 Inf  2.131  0.1324 
##  SafeVsRisky     0.336 0.189 Inf  1.771  0.3062 
## 
## Results are averaged over the levels of: antID 
## P value adjustment: bonferroni method for 4 tests
```

```
exp2p<-subset(exp2,exp2$visit<9) #just remove tests
exp2p <- droplevels(exp2p)
```

#### Back to the nest

```
mpExp2<-glmer(pherbk~visit*mol+(1|colony/antID),data=exp2,family=poisson,
              glmerControl(optimizer="bobyqa", optCtrl = list(maxfun = 100000)))
simres<-simulateResiduals(mpExp2) #standard seed for random values is 123
plot(simres, asFactor=T)
```

the model is zero inflated. let’s remodel

```
mpExp2 <- zeroinfl(pherbk ~ visit*mol + 1 | colony/antID, data = exp2)
```

```
## Error in optim(fn = loglikfun, gr = gradfun, par = c(start$count, start$zero, : valore non finito fornito da optim
```

does not work. I will remove colony from random effect.

```
mpExp2 <- zeroinfl(pherbk ~ visit*mol + 1 | antID, data = exp2)

Anova(mpExp2)
```

```
## Analysis of Deviance Table (Type II tests)
## 
## Response: pherbk
##           Df   Chisq Pr(>Chisq)    
## visit      1  10.249   0.001368 ** 
## mol        2 133.424  < 2.2e-16 ***
## visit:mol  2  11.339   0.003449 ** 
## ---
## Signif. codes:  0 '***' 0.001 '**' 0.01 '*' 0.05 '.' 0.1 ' ' 1
```

```
meanobj<-emmeans(mpExp2,~visit*mol,type="response")
contrast(meanobj,list(mol0.1vs0.55=c(1,-1,0),
                      mol0.1vs1.5=c(1,0,-1),
                      mol0.55vs1.5=c(0,1,-1),
                      SafeVsRisky=c(-0.5,1,-0.5)),
         adjust="bonferroni")
```

```
##  contrast     estimate    SE  df z.ratio p.value
##  mol0.1vs0.55   -2.684 0.170 Inf -15.742 <.0001 
##  mol0.1vs1.5    -3.474 0.204 Inf -17.000 <.0001 
##  mol0.55vs1.5   -0.790 0.191 Inf  -4.144 0.0001 
##  SafeVsRisky     0.947 0.149 Inf   6.341 <.0001 
## 
## Results are averaged over the levels of: antID 
## P value adjustment: bonferroni method for 4 tests
```

### Exp3

#### To the drop

```
exp3$visit<-as.numeric(exp3$visit)

mpExp3<-glmer(phergo~visit*mol+(1|colony/antID),data=exp3,family=poisson, glmerControl(optimizer="bobyqa", optCtrl = list(maxfun = 100000)))
```

```
## boundary (singular) fit: see ?isSingular
```

```
simres<-simulateResiduals(mpExp3) #standard seed for random values is 123
plot(simres, asFactor=T)
```

```
Anova(mpExp3)
```

```
## Analysis of Deviance Table (Type II Wald chisquare tests)
## 
## Response: phergo
##             Chisq Df Pr(>Chisq)    
## visit      0.2874  1  0.5919150    
## mol       16.1337  2  0.0003138 ***
## visit:mol  3.7145  2  0.1560975    
## ---
## Signif. codes:  0 '***' 0.001 '**' 0.01 '*' 0.05 '.' 0.1 ' ' 1
```

```
meanobj<-emmeans(mpExp3,~visit*mol,type="response")
contrast(meanobj,list(mol0.1vs0.3=c(1,-1,0),
                      mol0.1vs0.9=c(1,0,-1),
                      mol0.3vs0.9=c(0,1,-1),
                      SafeVsRisky=c(-0.5,1,-0.5)),
         adjust="bonferroni")
```

```
##  contrast     ratio    SE  df z.ratio p.value
##  mol0.1vs0.3  0.477 0.174 Inf -2.032  0.1687 
##  mol0.1vs0.9  4.981 3.453 Inf  2.317  0.0820 
##  mol0.3vs0.9 10.444 6.501 Inf  3.769  0.0007 
##  SafeVsRisky  4.679 1.751 Inf  4.124  0.0001 
## 
## P value adjustment: bonferroni method for 4 tests 
## Tests are performed on the log scale
```

```
exp3p<-subset(exp3,exp3$visit<9) #just remove tests
exp3p <- droplevels(exp3p)
```

#### Back to the nest

```
mpExp3<-glmer(pherbk~visit*mol+(1|colony/antID),data=exp3,family=poisson,
              glmerControl(optimizer="bobyqa", optCtrl = list(maxfun = 100000)))
simres<-simulateResiduals(mpExp3) #standard seed for random values is 123
plot(simres, asFactor=T)
```

the model is zero inflated. let’s remodel

```
mpExp3 <- zeroinfl(pherbk ~ visit*mol + 1 | colony/antID, data = exp3)
```

```
## Error in optim(fn = loglikfun, gr = gradfun, par = c(start$count, start$zero, : valore non finito fornito da optim
```

does not work. I will remove colony from random effect.

```
mpExp3 <- zeroinfl(pherbk ~ visit*mol + 1 |antID, data = exp3)

Anova(mpExp3)
```

```
## Analysis of Deviance Table (Type II tests)
## 
## Response: pherbk
##           Df   Chisq Pr(>Chisq)   
## visit      1  0.7228   0.395222   
## mol        2 12.7128   0.001736 **
## visit:mol  2  9.4469   0.008884 **
## ---
## Signif. codes:  0 '***' 0.001 '**' 0.01 '*' 0.05 '.' 0.1 ' ' 1
```

```
meanobj<-emmeans(mpExp3,~visit*mol,type="response")
contrast(meanobj,list(mol0.1vs0.3=c(1,-1,0),
                      mol0.1vs0.9=c(1,0,-1),
                      mol0.3vs0.9=c(0,1,-1),
                      SafeVsRisky=c(-0.5,1,-0.5)),
         adjust="bonferroni")
```

```
##  contrast    estimate    SE  df z.ratio p.value
##  mol0.1vs0.3 -0.00968 0.380 Inf -0.025  1.0000 
##  mol0.1vs0.9 -0.52436 0.391 Inf -1.341  0.7201 
##  mol0.3vs0.9 -0.51469 0.144 Inf -3.569  0.0014 
##  SafeVsRisky -0.25250 0.211 Inf -1.199  0.9224 
## 
## Results are averaged over the levels of: antID 
## P value adjustment: bonferroni method for 4 tests
```

### Exp4

#### To the drop

```
exp4$visit<-as.numeric(exp4$visit)
exp4$mol<-as.factor(exp4$mol)

mpexp4<-glmer(phergo~visit*mol+(1|colony/antID),data=exp4,family=poisson, glmerControl(optimizer="bobyqa", optCtrl = list(maxfun = 100000)))
```

```
## Warning in checkConv(attr(opt, "derivs"), opt$par, ctrl = control$checkConv, :
## Model failed to converge with max|grad| = 0.0751206 (tol = 0.002, component 1)
```

```
## Warning in checkConv(attr(opt, "derivs"), opt$par, ctrl = control$checkConv, : Model is nearly unidentifiable: very large eigenvalue
##  - Rescale variables?
```

failed to converge, dropping random

```
mpexp4<-glm(phergo~visit*mol, data=exp4,family=poisson)

simres<-simulateResiduals(mpexp4) #standard seed for random values is 123
plot(simres, asFactor=T)
```

zero inflated, remodel

```
mpexp4 <- zeroinfl(phergo ~ visit*mol, data = exp4)
```

```
Anova(mpexp4)
```

```
## Analysis of Deviance Table (Type II tests)
## 
## Response: phergo
##           Df  Chisq Pr(>Chisq)
## visit      1 0.0380     0.8455
## mol        2 2.6213     0.2696
## visit:mol  2 0.6906     0.7080
```

```
meanobj<-emmeans(mpexp4,~visit*mol,type="response")
contrast(meanobj,list(mol0.25vs0.5=c(1,-1,0),
                      mol0.25vs2.0=c(1,0,-1),
                      mol0.5vs2.0=c(0,1,-1),
                      SafeVsRisky=c(-0.5,1,-0.5)),
         adjust="bonferroni")
```

```
##  contrast     estimate    SE  df z.ratio p.value
##  mol0.25vs0.5   0.1427 0.135 Inf  1.059  1.0000 
##  mol0.25vs2.0   0.2192 0.151 Inf  1.450  0.5880 
##  mol0.5vs2.0    0.0765 0.126 Inf  0.607  1.0000 
##  SafeVsRisky   -0.0331 0.106 Inf -0.312  1.0000 
## 
## P value adjustment: bonferroni method for 4 tests
```

```
exp4p<-subset(exp4,exp4$visit<9) #just remove tests
exp4p <- droplevels(exp4p)
```

#### Back to the nest

```
mpexp4<-glmer(pherbk~visit*mol+(1|colony/antID),data=exp4,family=poisson,
              glmerControl(optimizer="bobyqa", optCtrl = list(maxfun = 100000)))
```

```
## boundary (singular) fit: see ?isSingular
```

```
simres<-simulateResiduals(mpexp4) #standard seed for random values is 123
plot(simres, asFactor=T)
```

the model is zero inflated. let’s remodel

```
mpexp4 <- zeroinfl(pherbk ~ visit*mol + 1 | colony/antID, data = exp4)
```

```
## Error in optim(fn = loglikfun, gr = gradfun, par = c(start$count, start$zero, : valore non finito fornito da optim
```

does not work. I will remove colony from random effect.

```
mpexp4 <- zeroinfl(pherbk ~ visit*mol + 1 |antID, data = exp4)

Anova(mpexp4)
```

```
## Analysis of Deviance Table (Type II tests)
## 
## Response: pherbk
##           Df    Chisq Pr(>Chisq)    
## visit      1   1.1532    0.28288    
## mol        2 121.1146    < 2e-16 ***
## visit:mol  2   4.8149    0.09004 .  
## ---
## Signif. codes:  0 '***' 0.001 '**' 0.01 '*' 0.05 '.' 0.1 ' ' 1
```

```
meanobj<-emmeans(mpexp4,~mol,type="response")
```

```
## NOTE: Results may be misleading due to involvement in interactions
```

```
contrast(meanobj,list(mol0.25vs0.5=c(1,-1,0),
                      mol0.25vs2.0=c(1,0,-1),
                      mol0.5vs2.0=c(0,1,-1),
                      SafeVsRisky=c(-0.5,1,-0.5)),
         adjust="bonferroni")
```

```
##  contrast     estimate    SE  df z.ratio p.value
##  mol0.25vs0.5   -1.078 0.191 Inf  -5.657 <.0001 
##  mol0.25vs2.0   -2.531 0.249 Inf -10.175 <.0001 
##  mol0.5vs2.0    -1.453 0.187 Inf  -7.768 <.0001 
##  SafeVsRisky    -0.187 0.142 Inf  -1.319 0.7492 
## 
## Results are averaged over the levels of: antID 
## P value adjustment: bonferroni method for 4 tests
```

### Exp5

#### To the drop

```
exp5$visit<-as.numeric(exp5$visit)
exp5$mol_feed <-paste(exp5$mol, exp5$feed) #this is needed since mol 0.25 is in both feeder and they need to be considered separately
exp5$mol_feed[exp5$mol_feed == "NA NA"] <- NA
exp5$mol_feed <- as.factor(exp5$mol_feed)

mpexp5<-glmer(phergo~visit*mol_feed+(1|colony/antID),data=exp5,family=poisson, glmerControl(optimizer="bobyqa", optCtrl = list(maxfun = 100000)))
simres<-simulateResiduals(mpexp5) #standard seed for random values is 123
plot(simres, asFactor=T)
```

the model is zero inflated. let’s remodel

```
mpexp5 <- zeroinfl(phergo ~ visit*mol_feed + 1 | colony/antID, data = exp5)
```

```
## Error in optim(fn = loglikfun, gr = gradfun, par = c(start$count, start$zero, : valore non finito fornito da optim
```

does not work. I will remove random effect.

```
mpexp5 <- zeroinfl(phergo ~ visit*mol_feed + 1 | antID, data = exp5)

Anova(mpexp5)
```

```
## Analysis of Deviance Table (Type II tests)
## 
## Response: phergo
##                Df  Chisq Pr(>Chisq)   
## visit           1 7.2851   0.006953 **
## mol_feed        2 0.2882   0.865789   
## visit:mol_feed  2 5.7211   0.057238 . 
## ---
## Signif. codes:  0 '***' 0.001 '**' 0.01 '*' 0.05 '.' 0.1 ' ' 1
```

```
trendobj<-emtrends(mpexp5,'mol_feed','visit')
test(trendobj)
```

```
##  mol_feed   visit.trend     SE  df z.ratio p.value
##  0.25 risky      0.0798 0.0454 Inf 1.760   0.0785 
##  0.25 safe       0.0180 0.0451 Inf 0.400   0.6892 
##  1.5 risky       0.1364 0.0306 Inf 4.455   <.0001 
## 
## Results are averaged over the levels of: antID
```

```
meanobj<-emmeans(mpexp5,~visit*mol_feed,type="response")
contrast(meanobj,list(mol0.25risky_vs_0.25safe=c(1,-1,0),
                      mol0.25risky_vs_1.5risky=c(1,0,-1),
                      mol0.25safe_vs_1.5risky=c(0,1,-1),
                      SafeVsRisky=c(-0.5,1,-0.5)),
         adjust="bonferroni")
```

```
##  contrast                 estimate    SE  df z.ratio p.value
##  mol0.25risky_vs_0.25safe  -0.0546 0.135 Inf -0.405  1.0000 
##  mol0.25risky_vs_1.5risky   0.2413 0.152 Inf  1.587  0.4503 
##  mol0.25safe_vs_1.5risky    0.2959 0.143 Inf  2.076  0.1517 
##  SafeVsRisky                0.1753 0.116 Inf  1.509  0.5256 
## 
## Results are averaged over the levels of: antID 
## P value adjustment: bonferroni method for 4 tests
```

```
exp5p<-subset(exp5,exp5$visit<9) #just remove tests
exp5p <- droplevels(exp5p)
```

#### Back to the nest

```
mpexp5<-glmer(pherbk~visit*mol_feed+(1|colony/antID),data=exp5,family=poisson,
              glmerControl(optimizer="bobyqa", optCtrl = list(maxfun = 100000)))
simres<-simulateResiduals(mpexp5) #standard seed for random values is 123
plot(simres, asFactor=T)
```

the model is zero inflated. let’s remodel

```
mpexp5 <- zeroinfl(pherbk ~ visit*mol_feed + 1 | colony/antID, data = exp5)
```

```
## Error in optim(fn = loglikfun, gr = gradfun, par = c(start$count, start$zero, : valore non finito fornito da optim
```

does not work. I will remove colony from random effect.

```
mpexp5 <- zeroinfl(pherbk ~ visit*mol_feed + 1 |antID, data = exp5)

Anova(mpexp5)
```

```
## Analysis of Deviance Table (Type II tests)
## 
## Response: pherbk
##                Df   Chisq Pr(>Chisq)    
## visit           1  1.8572    0.17294    
## mol_feed        2 38.6628  4.022e-09 ***
## visit:mol_feed  2  8.1910    0.01665 *  
## ---
## Signif. codes:  0 '***' 0.001 '**' 0.01 '*' 0.05 '.' 0.1 ' ' 1
```

```
meanobj<-emmeans(mpexp5,~visit*mol_feed,type="response")
contrast(meanobj,list(mol0.25risky_vs_0.25safe=c(1,-1,0),
                      mol0.25risky_vs_1.5risky=c(1,0,-1),
                      mol0.25safe_vs_1.5risky=c(0,1,-1),
                      SafeVsRisky=c(-0.5,1,-0.5)),
         adjust="bonferroni")
```

```
##  contrast                 estimate    SE  df z.ratio p.value
##  mol0.25risky_vs_0.25safe   -0.361 0.184 Inf -1.956  0.2016 
##  mol0.25risky_vs_1.5risky   -1.186 0.193 Inf -6.151  <.0001 
##  mol0.25safe_vs_1.5risky    -0.825 0.151 Inf -5.466  <.0001 
##  SafeVsRisky                -0.232 0.138 Inf -1.680  0.3722 
## 
## Results are averaged over the levels of: antID 
## P value adjustment: bonferroni method for 4 tests
```

```
trendobj<-emtrends(mpexp5,'mol_feed','visit')
test(trendobj)
```

```
##  mol_feed   visit.trend     SE  df z.ratio p.value
##  0.25 risky    -0.15779 0.0495 Inf -3.187  0.0014 
##  0.25 safe     -0.08584 0.0482 Inf -1.781  0.0749 
##  1.5 risky     -0.00125 0.0392 Inf -0.032  0.9745 
## 
## Results are averaged over the levels of: antID
```

### graph together

now I will plot the pheromone deposition all together for the three experiments

```
library(cowplot)
```

```
## 
## ********************************************************
```

```
## Note: As of version 1.0.0, cowplot does not change the
```

```
##   default ggplot2 theme anymore. To recover the previous
```

```
##   behavior, execute:
##   theme_set(theme_cowplot())
```

```
## ********************************************************
```

```
exp1p <- subset(exp1, exp1$visit<9)
exp2p <- subset(exp2, exp2$visit<9)
exp3p <- subset(exp3, exp3$visit<9)
exp4p <- subset(exp4, exp4$visit<9)
exp5p <- subset(exp5, exp5$visit<9)

e1g <- ggplot(exp1p, aes(x=mol, y=phergo, fill=feed, color = feed))+
  geom_boxplot()+
  geom_jitter(width = 0.02,height=0.3, shape=1)+
  scale_fill_manual(name="molarity", values = c("#E69F00", "#56B4E9"))+
  scale_color_manual(name="molarity",values= c("#863F00", "#005489"))+
  ylab("Pheromone deposited to the feeder")+
  ylim(0,20)+
  theme_light()+
  theme(axis.text.x = element_blank(),
    axis.text.y = element_text(size=18,colour="black"),
    axis.title.x = element_blank(),
    axis.title.y = element_text(size=16),
    plot.title = element_text(size=18),
    legend.position="none")

e1b <- ggplot(exp1p, aes(x=mol, y=pherbk, fill=feed, color = feed))+
  geom_boxplot()+
  geom_jitter(width = 0.02,height=0.3, shape=1)+
  scale_fill_manual(name="molarity", values = c("#E69F00", "#56B4E9"))+
  scale_color_manual(name="molarity",values= c("#863F00", "#005489"))+
  ylab("Pheromone deposited back to the nest")+
  ylim(0,20)+
  theme_light()+
  theme(axis.text.x = element_text(size=18,colour="black"),
    axis.text.y = element_text(size=18,colour="black"),
    axis.title.x = element_text(size=16),
    axis.title.y = element_text(size=16),
    plot.title = element_text(size=18),
    legend.position="none")

e2g <- ggplot(exp2p, aes(x=mol, y=phergo, fill=feed, color = feed))+
  geom_boxplot()+
  geom_jitter(width = 0.02,height=0.3, shape=1)+
  scale_fill_manual(name="molarity", values = c("#E69F00", "#56B4E9"))+
  scale_color_manual(name="molarity",values= c("#863F00", "#005489"))+
  ylim(0,20)+
  theme_light()+
  theme(axis.text.x = element_blank(),
    axis.text.y = element_blank(),
    axis.title.x = element_blank(),
    axis.title.y = element_blank(),
    plot.title = element_text(size=18),
    legend.position="none")

e2b <- ggplot(exp2p, aes(x=mol, y=pherbk, fill=feed, color = feed))+
  geom_boxplot()+
  geom_jitter(width = 0.02,height=0.3, shape=1)+
  scale_fill_manual(name="molarity", values = c("#E69F00", "#56B4E9"))+
  scale_color_manual(name="molarity",values= c("#863F00", "#005489"))+
  ylim(0,20)+
  theme_light()+
  theme(axis.text.x = element_text(size=18,colour="black"),
    axis.text.y = element_blank(),
    axis.title.x = element_text(size=16),
    axis.title.y = element_blank(),
    plot.title = element_text(size=18),
    legend.position="none")

e3g <- ggplot(exp3p, aes(x=mol, y=phergo, fill=feed, color = feed))+
  geom_boxplot()+
  geom_jitter(width = 0.02,height=0.3, shape=1)+
  scale_fill_manual(name="molarity", values = c("#E69F00", "#56B4E9"))+
  scale_color_manual(name="molarity",values= c("#863F00", "#005489"))+
  ylab("Pheromone deposited to the feeder")+
  ylim(0,20)+
  theme_light()+
  theme(axis.text.x = element_blank(),
    axis.text.y = element_blank(),
    axis.title.x = element_blank(),
    axis.title.y = element_blank(),
    plot.title = element_text(size=18),
    legend.position="none")

e3b <- ggplot(exp3p, aes(x=mol, y=pherbk, fill=feed, color = feed))+
  geom_boxplot()+
  geom_jitter(width = 0.02,height=0.3, shape=1)+
  scale_fill_manual(name="molarity", values = c("#E69F00", "#56B4E9"))+
  scale_color_manual(name="molarity",values= c("#863F00", "#005489"))+
  ylab("Pheromone deposited back to the nest")+
  ylim(0,20)+
  theme_light()+
  theme(axis.text.x = element_text(size=18,colour="black"),
    axis.text.y = element_blank(),
    axis.title.x = element_text(size=16),
    axis.title.y = element_blank(),
    plot.title = element_text(size=18),
    legend.position="none")

e4g <- ggplot(exp4p, aes(x=mol, y=phergo, fill=feed, color = feed))+
  geom_boxplot()+
  geom_jitter(width = 0.02,height=0.3, shape=1)+
  scale_fill_manual(name="molarity", values = c("#E69F00", "#56B4E9"))+
  scale_color_manual(name="molarity",values= c("#863F00", "#005489"))+
  ylab("Pheromone deposited to the feeder")+
  ylim(0,20)+
  theme_light()+
  theme(axis.text.x = element_blank(),
    axis.text.y = element_blank(),
    axis.title.x = element_blank(),
    axis.title.y = element_blank(),
    plot.title = element_text(size=18),
    legend.position="none")

e4b <- ggplot(exp4p, aes(x=mol, y=pherbk, fill=feed, color = feed))+
  geom_boxplot()+
  geom_jitter(width = 0.02,height=0.3, shape=1)+
  scale_fill_manual(name="molarity", values = c("#E69F00", "#56B4E9"))+
  scale_color_manual(name="molarity",values= c("#863F00", "#005489"))+
  ylab("Pheromone deposited back to the nest")+
  ylim(0,20)+
  theme_light()+
  theme(axis.text.x = element_text(size=18,colour="black"),
    axis.text.y = element_blank(),
    axis.title.x = element_text(size=16),
    axis.title.y = element_blank(),
    plot.title = element_text(size=18),
    legend.position="none")

e5g <- ggplot(exp5p, aes(x=mol, y=phergo, fill=feed, color = feed))+
  geom_boxplot()+
  geom_point(shape=1, position=position_jitterdodge(0.02, jitter.height = 0.3, dodge.width = 0.8))+
  scale_fill_manual(name="molarity", values = c("#E69F00", "#56B4E9"))+
  scale_color_manual(name="molarity",values= c("#863F00", "#005489"))+
  ylab("Pheromone deposited to the feeder")+
  ylim(0,20)+
  theme_light()+
  theme(axis.text.x = element_blank(),
    axis.text.y = element_blank(),
    axis.title.x = element_blank(),
    axis.title.y = element_blank(),
    plot.title = element_text(size=18),
    legend.position="none")

e5b <- ggplot(exp5p, aes(x=mol, y=pherbk, fill=feed, color = feed))+
  geom_boxplot()+
  geom_point(shape=1, position=position_jitterdodge(0.02, jitter.height = 0.3, dodge.width = 0.8))+
  scale_fill_manual(name="molarity", values = c("#E69F00", "#56B4E9"))+
  scale_color_manual(name="molarity",values= c("#863F00", "#005489"))+
  ylab("Pheromone deposited back to the nest")+
  ylim(0,20)+
  theme_light()+
  theme(axis.text.x = element_text(size=18,colour="black"),
    axis.text.y = element_blank(),
    axis.title.x = element_text(size=16),
    axis.title.y = element_blank(),
    plot.title = element_text(size=18),
    legend.position="none")

toprow<- plot_grid(e1g,e2g,e3g,e4g,e5g, labels= c('A', 'C', 'E', 'G', 'I'), label_size = 18, ncol = 5)
```

```
## Warning: Removed 64 rows containing non-finite values (stat_boxplot).
```

```
## Warning: Removed 187 rows containing missing values (geom_point).
```

```
## Warning: Removed 69 rows containing non-finite values (stat_boxplot).
```

```
## Warning: Removed 184 rows containing missing values (geom_point).
```

```
## Warning: Removed 43 rows containing non-finite values (stat_boxplot).
```

```
## Warning: Removed 168 rows containing missing values (geom_point).
```

```
## Warning: Removed 64 rows containing non-finite values (stat_boxplot).
```

```
## Warning: Removed 271 rows containing missing values (geom_point).
```

```
## Warning: Removed 64 rows containing non-finite values (stat_boxplot).
```

```
## Warning: Removed 232 rows containing missing values (geom_point).
```

```
bottomrow<- plot_grid(e1b,e2b,e3b,e4b,e5b, labels= c('B', 'D', 'F', 'H', 'L'), label_size = 18, ncol = 5)
```

```
## Warning: Removed 1 rows containing non-finite values (stat_boxplot).
```

```
## Warning: Removed 171 rows containing missing values (geom_point).
```

```
## Warning: Removed 18 rows containing non-finite values (stat_boxplot).
```

```
## Warning: Removed 150 rows containing missing values (geom_point).
```

```
## Warning: Removed 5 rows containing non-finite values (stat_boxplot).
```

```
## Warning: Removed 117 rows containing missing values (geom_point).
```

```
## Warning: Removed 116 rows containing missing values (geom_point).
```

```
## Warning: Removed 2 rows containing non-finite values (stat_boxplot).
```

```
## Warning: Removed 184 rows containing missing values (geom_point).
```

```
plot_grid(toprow, bottomrow, ncol = 1)
```

# Supplemental pilot experiment

## Ant perception of 0.1,0.3,0.9

```
ctrl <- rbind(supp13,supp39)
ctrlmelted<-melt(ctrl, measure.vars = c("Firstchoice","Secondchoice"))

m0<-glmer(value~contrast*Visitnumber+(1|AntID),data=ctrlmelted,family=binomial,
          glmerControl(optimizer="bobyqa", optCtrl = list(maxfun = 100000000)))
Anova(m0)
```

```
## Analysis of Deviance Table (Type II Wald chisquare tests)
## 
## Response: value
##                       Chisq Df Pr(>Chisq)  
## contrast             3.5856  1    0.05828 .
## Visitnumber          1.4604  1    0.22687  
## contrast:Visitnumber 0.0242  1    0.87632  
## ---
## Signif. codes:  0 '***' 0.001 '**' 0.01 '*' 0.05 '.' 0.1 ' ' 1
```

no difference between visits

```
m1<-glmer(value~contrast*variable+(1|Colony/AntID),data=ctrlmelted,family=binomial,
          glmerControl(optimizer="bobyqa", optCtrl = list(maxfun = 100000000)))
```

```
## boundary (singular) fit: see ?isSingular
```

```
e<-emmeans(m1,~contrast*variable,type="response")
test(e,adjust="bonferroni")
```

```
##  contrast variable      prob     SE  df z.ratio p.value
##  0.1vs0.3 Firstchoice  0.860 0.0569 Inf 3.844   0.0005 
##  0.3vs0.9 Firstchoice  0.648 0.0953 Inf 1.461   0.5764 
##  0.1vs0.3 Secondchoice 0.870 0.0539 Inf 3.987   0.0003 
##  0.3vs0.9 Secondchoice 0.724 0.0850 Inf 2.264   0.0942 
## 
## P value adjustment: bonferroni method for 4 tests 
## Tests are performed on the logit scale
```

## Discriminate three drops

```
melted <- melt(suppthreemol, measure.vars = c("Firstchoice","Secondchoice"))
m0<-glmer(value~Visitnumber+(1|Colony/AntID),data=melted,family=binomial,
          glmerControl(optimizer="bobyqa", optCtrl = list(maxfun = 100000000)))
Anova(m0)
```

```
## Analysis of Deviance Table (Type II Wald chisquare tests)
## 
## Response: value
##              Chisq Df Pr(>Chisq)  
## Visitnumber 4.5959  1    0.03205 *
## ---
## Signif. codes:  0 '***' 0.001 '**' 0.01 '*' 0.05 '.' 0.1 ' ' 1
```

difference between visits

```
m1<-glmer(value~variable+(1|Colony/AntID),data=melted,family=binomial,
          glmerControl(optimizer="bobyqa", optCtrl = list(maxfun = 100000000)))
Anova(m1)
```

```
## Analysis of Deviance Table (Type II Wald chisquare tests)
## 
## Response: value
##           Chisq Df Pr(>Chisq)
## variable 0.1038  1     0.7473
```

no difference between first and last choice. I will just look at all the percentages together.

```
melted$Visitnumber<-as.factor(melted$Visitnumber)
m2<-glmer(value~variable*Visitnumber+(1|Colony/AntID),data=melted,family=binomial,
          glmerControl(optimizer="bobyqa", optCtrl = list(maxfun = 100000000)))
```

```
## Warning in checkConv(attr(opt, "derivs"), opt$par, ctrl = control$checkConv, : Model is nearly unidentifiable: large eigenvalue ratio
##  - Rescale variables?
```

```
e<-emmeans(m2,~variable*Visitnumber,type="response")
e
```

```
##  variable     Visitnumber      prob         SE  df asymp.LCL asymp.UCL
##  Firstchoice  10          1.0000000 0.00000014 Inf 0.0000000 1.0000000
##  Secondchoice 10          1.0000000 0.00000021 Inf 0.0000000 1.0000000
##  Firstchoice  11          0.9580370 0.05009378 Inf 0.6650515 0.9962051
##  Secondchoice 11          0.9580370 0.05009388 Inf 0.6650504 0.9962052
##  Firstchoice  12          0.9580370 0.05009380 Inf 0.6650513 0.9962052
##  Secondchoice 12          0.9580370 0.05009388 Inf 0.6650504 0.9962052
##  Firstchoice  13          0.9580370 0.05009367 Inf 0.6650526 0.9962051
##  Secondchoice 13          0.9580370 0.05009387 Inf 0.6650506 0.9962052
##  Firstchoice  14          0.9074061 0.08321647 Inf 0.5844706 0.9855653
##  Secondchoice 14          0.8481658 0.11228538 Inf 0.5028425 0.9686048
## 
## Confidence level used: 0.95 
## Intervals are back-transformed from the logit scale
```

I have a 100% probability of choosing the highest molarity for the first trial, the percentage decrease with subsequent, but it remains very high.

## fed on 1.5 risk in losses

### Preliminary questions

#### initial vs. final

first, I want to know if initial and final choice differ

```
fsdiff<-melt(supploss, measure.vars = c("firstchoicesafe","endchoicesafe"))

mdiff<-glmer(value~variable+(1|colony/antID),data=fsdiff,family=binomial)
```

```
## boundary (singular) fit: see ?isSingular
```

```
Anova(mdiff)
```

```
## Analysis of Deviance Table (Type II Wald chisquare tests)
## 
## Response: value
##           Chisq Df Pr(>Chisq)
## variable 0.0952  1     0.7577
```

```
e<-emmeans(mdiff, ~variable, type="response")
pairs(e)
```

```
##  contrast                        odds.ratio    SE  df z.ratio p.value
##  firstchoicesafe / endchoicesafe        1.1 0.339 Inf 0.309   0.7577 
## 
## Tests are performed on the log odds ratio scale
```

there is no difference between primary and secondary choice, I will now on only use the primary for further analysis

#### vistits n.

now, I want to know if the visits differ from one another

```
supploss$visit<-as.numeric(supploss$visit)
mvisdiff<-glmer(firstchoicesafe~visit+(1|colony/antID),data=supploss,family="binomial",
                glmerControl(optimizer="bobyqa", optCtrl = list(maxfun = 1000000000)))
```

```
## boundary (singular) fit: see ?isSingular
```

```
Anova(mvisdiff)
```

```
## Analysis of Deviance Table (Type II Wald chisquare tests)
## 
## Response: firstchoicesafe
##        Chisq Df Pr(>Chisq)  
## visit 4.6318  1    0.03138 *
## ---
## Signif. codes:  0 '***' 0.001 '**' 0.01 '*' 0.05 '.' 0.1 ' ' 1
```

```
summary(mvisdiff)
```

```
## Generalized linear mixed model fit by maximum likelihood (Laplace
##   Approximation) [glmerMod]
##  Family: binomial  ( logit )
## Formula: firstchoicesafe ~ visit + (1 | colony/antID)
##    Data: supploss
## Control: glmerControl(optimizer = "bobyqa", optCtrl = list(maxfun = 1e+09))
## 
##      AIC      BIC   logLik deviance df.resid 
##    193.9    206.9    -93.0    185.9      185 
## 
## Scaled residuals: 
##     Min      1Q  Median      3Q     Max 
## -2.4228  0.2194  0.2921  0.3888  0.8847 
## 
## Random effects:
##  Groups       Name        Variance Std.Dev.
##  antID:colony (Intercept) 2.385    1.544   
##  colony       (Intercept) 0.000    0.000   
## Number of obs: 189, groups:  antID:colony, 63; colony, 4
## 
## Fixed effects:
##             Estimate Std. Error z value Pr(>|z|)   
## (Intercept)   7.5728     2.7825   2.722   0.0065 **
## visit        -0.5722     0.2659  -2.152   0.0314 * 
## ---
## Signif. codes:  0 '***' 0.001 '**' 0.01 '*' 0.05 '.' 0.1 ' ' 1
## 
## Correlation of Fixed Effects:
##       (Intr)
## visit -0.991
## convergence code: 0
## boundary (singular) fit: see ?isSingular
```

the percentage of ants going for safe decreases with successive visits. this means that more and more ants after not finding the sugar drop start doing a random search. I will from now on only observe the first visit, being it a clearer indication of choice

### modeling

now to the actual model. I drop antID because I kept only one observation for each ant

```
supplosssing<-subset(supploss,supploss$visit==9)

mExp1<-glmer(firstchoicesafe~firstfeed*firstrisk+(1|antID),data=supplosssing,family="binomial",
             glmerControl(optimizer="bobyqa", optCtrl = list(maxfun = 1000000)))
```

```
## boundary (singular) fit: see ?isSingular
```

```
simres<-simulateResiduals(mExp1) #standard seed for random values is 123
plot(simres, asFactor=T)
```

model is good here. it says nearly unidentifiable. probably I have complete separation of one data point, like 100% prob for one group. let’s go on

```
Anova(mExp1)
```

```
## Analysis of Deviance Table (Type II Wald chisquare tests)
## 
## Response: firstchoicesafe
##                      Chisq Df Pr(>Chisq)
## firstfeed           1.1778  1     0.2778
## firstrisk           0.9249  1     0.3362
## firstfeed:firstrisk 0.0001  1     0.9931
```

no effect of any of the factors, so I will redo the model without factors

```
mExp1<-glmer(firstchoicesafe~+(1|antID),data=supplosssing,family="binomial",
             glmerControl(optimizer="bobyqa", optCtrl = list(maxfun = 1000000)))
```

```
## boundary (singular) fit: see ?isSingular
```

```
simres<-simulateResiduals(mExp1) #standard seed for random values is 123
plot(simres, asFactor=T)
```

```
meanobj <- emmeans(mExp1,~1, type="response")
(test(meanobj))
```

```
##  1        prob     SE  df z.ratio p.value
##  overall 0.825 0.0478 Inf 4.681   <.0001 
## 
## Tests are performed on the logit scale
```

ants prefer the safe **82%**.

# Session info

```
## R version 4.0.5 (2021-03-31)
## Platform: x86_64-pc-linux-gnu (64-bit)
## Running under: Manjaro Linux
## 
## Matrix products: default
## BLAS:   /usr/lib/libblas.so.3.9.1
## LAPACK: /usr/lib/liblapack.so.3.9.1
## 
## locale:
##  [1] LC_CTYPE=it_IT.UTF-8       LC_NUMERIC=C              
##  [3] LC_TIME=it_IT.UTF-8        LC_COLLATE=it_IT.UTF-8    
##  [5] LC_MONETARY=it_IT.UTF-8    LC_MESSAGES=it_IT.UTF-8   
##  [7] LC_PAPER=it_IT.UTF-8       LC_NAME=C                 
##  [9] LC_ADDRESS=C               LC_TELEPHONE=C            
## [11] LC_MEASUREMENT=it_IT.UTF-8 LC_IDENTIFICATION=C       
## 
## attached base packages:
## [1] stats     graphics  grDevices utils     datasets  methods   base     
## 
## other attached packages:
##  [1] cowplot_1.0.0  nonnest2_0.5-5 plyr_1.8.6     pscl_1.5.5     ggplot2_3.3.2 
##  [6] reshape2_1.4.4 emmeans_1.4.8  car_3.0-8      carData_3.0-4  DHARMa_0.3.2.0
## [11] lme4_1.1-23    Matrix_1.3-2   knitr_1.29     readxl_1.3.1  
## 
## loaded via a namespace (and not attached):
##  [1] Rcpp_1.0.5         mvtnorm_1.1-1      lattice_0.20-41    zoo_1.8-8         
##  [5] digest_0.6.25      foreach_1.5.0      R6_2.4.1           cellranger_1.1.0  
##  [9] stats4_4.0.5       evaluate_0.14      coda_0.19-3        highr_0.8         
## [13] pillar_1.4.6       rlang_0.4.7        curl_4.3           multcomp_1.4-13   
## [17] minqa_1.2.4        data.table_1.12.8  nloptr_1.2.2.2     gap_1.2.2         
## [21] pbivnorm_0.6.0     rmarkdown_2.3      labeling_0.3       splines_4.0.5     
## [25] statmod_1.4.34     stringr_1.4.0      foreign_0.8-81     munsell_0.5.0     
## [29] compiler_4.0.5     xfun_0.15          pkgconfig_2.0.3    mnormt_2.0.2      
## [33] tmvnsim_1.0-2      htmltools_0.5.0    tidyselect_1.1.0   tibble_3.0.3      
## [37] rio_0.5.16         codetools_0.2-18   withr_2.2.0        crayon_1.3.4      
## [41] dplyr_1.0.0        MASS_7.3-53.1      grid_4.0.5         nlme_3.1-152      
## [45] xtable_1.8-4       gtable_0.3.0       lifecycle_0.2.0    magrittr_1.5      
## [49] scales_1.1.1       zip_2.0.4          estimability_1.3   stringi_1.4.6     
## [53] farver_2.0.3       ellipsis_0.3.1     vctrs_0.3.2        generics_0.0.2    
## [57] boot_1.3-27        sandwich_2.5-1     openxlsx_4.1.5     TH.data_1.0-10    
## [61] iterators_1.0.12   tools_4.0.5        forcats_0.5.0      CompQuadForm_1.4.3
## [65] glue_1.4.1         purrr_0.3.4        hms_0.5.3          abind_1.4-5       
## [69] survival_3.2-3     yaml_2.2.1         colorspace_1.4-1   lavaan_0.6-8      
## [73] haven_2.3.1
```
